# Molecular insights into substrate recognition and catalytic mechanism of the chaperone and FKBP peptidyl-prolyl isomerase SlyD

**DOI:** 10.1186/s12915-016-0300-3

**Published:** 2016-09-23

**Authors:** Esben M. Quistgaard, Ulrich Weininger, Yonca Ural-Blimke, Kristofer Modig, Pär Nordlund, Mikael Akke, Christian Löw

**Affiliations:** 1 Department of Medical Biochemistry and Biophysics, Karolinska Institutet, Scheeles väg 2, SE-17177 Stockholm, Sweden; 2 Centre for Structural Systems Biology (CSSB), DESY and European Molecular Biology Laboratory Hamburg, Notkestrasse 85, D-22603 Hamburg, Germany; 3 Department of Biophysical Chemistry, Center for Molecular Protein Science, Lund University, PO Box 124, SE-221 00 Lund, Sweden; 4 School of Biological Sciences, Nanyang Technological University, 639798 Singapore, Singapore

**Keywords:** Peptidyl-prolyl isomerase (PPIase), FK506-binding protein (FKBP), Chaperone, Protein folding, Proline, Beta-turn, FK506, SlyD, NMR, X-ray crystal structure

## Abstract

**Background:**

Peptidyl-prolyl isomerases (PPIases) catalyze cis/trans isomerization of peptidyl-prolyl bonds, which is often rate-limiting for protein folding. SlyD is a two-domain enzyme containing both a PPIase FK506-binding protein (FKBP) domain and an insert-in-flap (IF) chaperone domain. To date, the interactions of these domains with unfolded proteins have remained rather obscure, with structural information on binding to the FKBP domain being limited to complexes involving various inhibitor compounds or a chemically modified tetrapeptide.

**Results:**

We have characterized the binding of 15-residue-long unmodified peptides to SlyD from *Thermus thermophilus* (TtSlyD) in terms of binding thermodynamics and enzyme kinetics through the use of isothermal titration calorimetry, nuclear magnetic resonance spectroscopy, and site-directed mutagenesis. We show that the affinities and enzymatic activity of TtSlyD towards these peptides are much higher than for the chemically modified tetrapeptides that are typically used for activity measurements on FKBPs. In addition, we present a series of crystal structures of TtSlyD with the inhibitor FK506 bound to the FKBP domain, and with 15-residue-long peptides bound to either one or both domains, which reveals that substrates bind in a highly adaptable fashion to the IF domain through β-strand augmentation, and can bind to the FKBP domain as both types VIa1 and VIb-like cis-proline β-turns. Our results furthermore provide important clues to the catalytic mechanism and support the notion of inter-domain cross talk.

**Conclusions:**

We found that 15-residue-long unmodified peptides can serve as better substrate mimics for the IF and FKBP domains than chemically modified tetrapeptides. We furthermore show how such peptides are recognized by each of these domains in TtSlyD, and propose a novel general model for the catalytic mechanism of FKBPs that involves C-terminal rotation around the peptidyl-prolyl bond mediated by stabilization of the twisted transition state in the hydrophobic binding site.

**Electronic supplementary material:**

The online version of this article (doi:10.1186/s12915-016-0300-3) contains supplementary material, which is available to authorized users.

## Background

Peptide bonds are planar with ω dihedral angles of either ~0° (*cis* form) or ~180° (*trans* form). Due to unfavorable steric and electronic effects, the *cis* form is by far the least favored, except for peptidyl-prolyl bonds where the unique N-alkylation of proline markedly reduces the energy difference between the two conformations [1, 2]. The *cis* form is therefore much more commonly observed for prolines than for any other residues [3]. In folded proteins, prolines are predominantly found in β-turns and other loop elements [3, 4], where the *cis* and *trans* isoforms have different effects on the structure. Although both isoforms can be found in type IV β-turns (a category with lax geometry requirements), the *trans* form specifically favors more narrowly defined types of turns, for example, I, II, and VIII, whereas the *cis* form is required for types VIa1, VIa2, and VIb [5, 6]. Protein folding requires that each proline in the sequence adopts the isoform compatible with the native fold [7, 8]. However, spontaneous *cis*/*trans* isomerization occurs very slowly due to the high energy barrier imposed by the partial double bond character of the peptide bond. Indeed, the isomerization correlation time typically falls in the seconds to minutes time regime [7]. Nature has therefore evolved three families of peptidyl-prolyl isomerases (PPIases) to facilitate *cis*/*trans* isomerization: FK506-binding proteins (FKBPs), cyclophilins, and parvulins [8, 9]. These enzymes presumably all function by stabilizing the transition state, resulting in an effective rate constant for the catalyzed reaction of up to 10^8^ M^−1^s^−1^ [9], but their mechanisms are not well understood [9, 10].

The first FKBP to be discovered was human FKBP12, which was identified as a binding partner of the immunosuppressive macrolide lactone FK506, hence the name of the family [11, 12]. Since then, it has become clear that FKBPs are widespread in all branches of life [9]. FKBPs often have additional chaperone or protein–protein interaction domains [8, 13]. A particularly well-studied example is SlyD [14]. This protein belongs to a prokaryotic subfamily, characterized by having an insert-in-flap (IF) chaperone domain inserted into the FKBP domain in place of the so-called flap loop (also known as the 80’s loop) found in FKBP12 and many other FKBPs [15], which both enables it to function as an efficient chaperone [16–19] and increases its PPIase activity towards partially folded protein substrates by as much as 100–200 fold [17, 20, 21]. The enzymatic activity of FKBPs has been studied in several ways, with the most popular method being a spectrophotometric assay that utilizes the modified tetrapeptide substrate analogue succinyl-Ala-Leu-Pro-Phe-4-nitroanilide (suc-ALPF-pNA) or variants thereof [8]. Structures have been determined for numerous FKBPs in both the apo and inhibitor-bound forms. However, to the best of our knowledge, only two structures have been obtained with a bound peptide, which in both cases is suc-ALPF-pNA [17, 22]. While chemically modified tetrapeptides are well suited for studying the effects of the residues neighboring the proline, they are not ideal substrate mimics, because they bind to *Escherichia coli* SlyD with much lower affinity than refolding protein substrates [23]. The low affinity of these peptides probably relates to the smaller interaction surface compared to protein substrates, but could also relate to their limited capacity to form naturally occurring structural elements, such as β-turns. Structural insights into how substrates interact with the IF domain have so far been based on a single structure of the SlyD homologue SlpA from *E. coli*, in which an uncleaved purification tag is bound at the substrate binding site of the IF domain [18].

In order to improve our understanding of the mechanism of SlyD and of FKBPs in general, we set out to analyze the kinetics, energetics, and structural basis for substrate binding and inhibition of SlyD from *Thermus thermophilus* (TtSlyD) using 15-residue-long unmodified peptides, which we reasoned would be better mimics of natural unfolded protein substrates than the traditionally used 4-nitroanilide tetrapeptides. Indeed, these long peptides display much improved binding affinity and enzymatic turnover compared to the tetrapeptides. The enzyme peptide complexes are fairly heterogeneous in their structural and energetic aspects, but common principles could be identified for both the IF and the FKBP domain. Our results shed new light on how substrates are recognized, and have enabled us to propose a model for the catalytic mechanism.

## Results

To investigate the mechanism of TtSlyD, we characterized its substrate binding and catalytic properties, as well as the three-dimensional structures of a number of TtSlyD:peptide complexes, through the use of isothermal titration calorimetry (ITC), nuclear magnetic resonance (NMR) spectroscopy, X-ray crystallography, and site-specific mutagenesis.

### Peptide binding studies

**Fig. 1:**
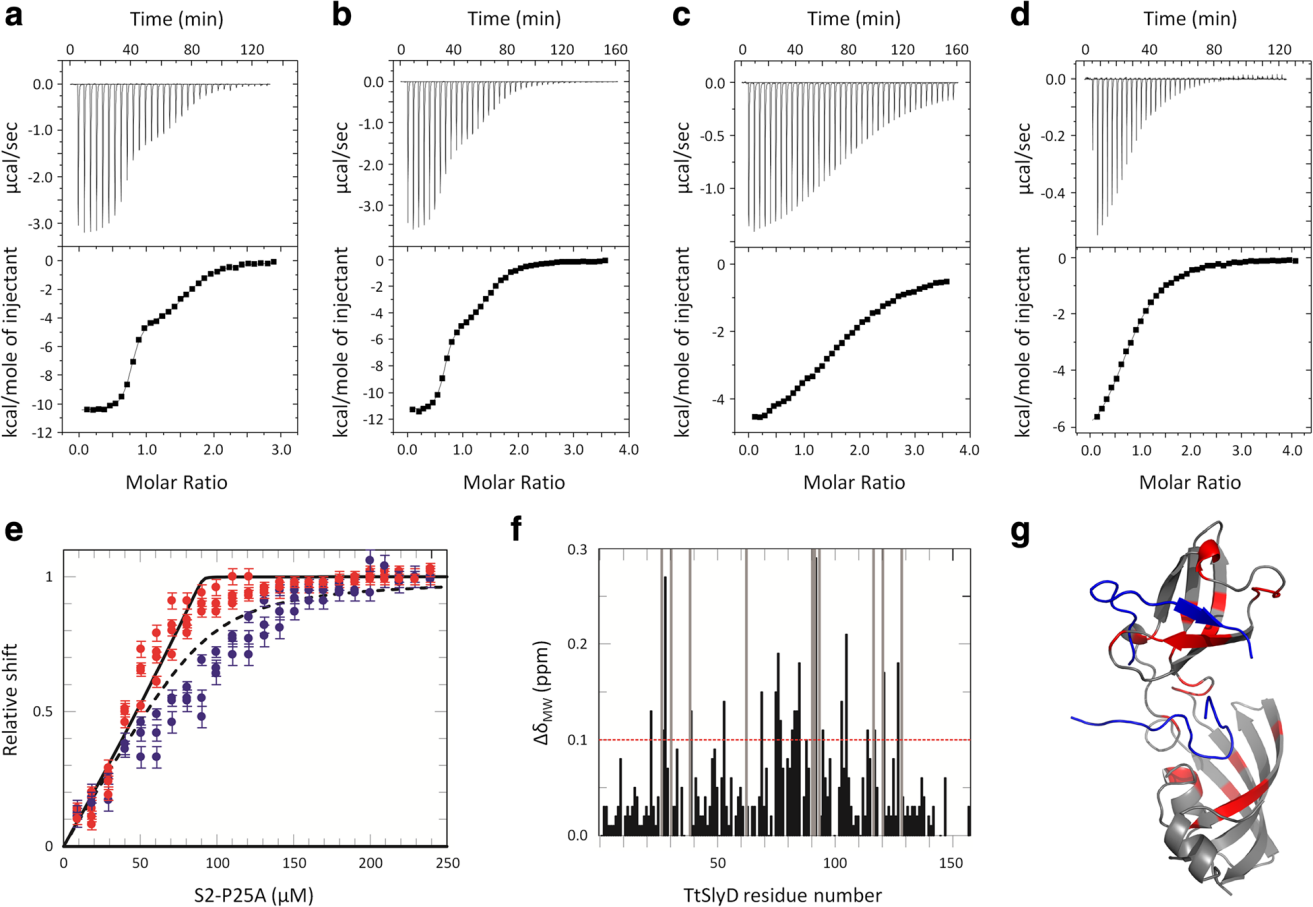
Isothermal titration calorimetry (ITC) and nuclear magnetic resonance (NMR) binding studies. Typical ITC data are shown for binding of peptides to TtSlyD: **a** Binding of the S2 peptide to full-length TtSlyD (TtSlyD_FL_). **b** Binding of the S2-long2 peptide to TtSlyD_FL_. **c** Binding of the S2-short6 peptide to TtSlyD_FL_. **d** Binding of the S2-W23A peptide to a TtSlyD construct in which the insert-in-flap (IF) domain is replaced by the flap loop from human FKBP12 (TtSlyD_ΔIF_). *Upper panels*: raw heat pulse data. *Lower panels*: Integrated heat changes upon binding plotted against the peptide/protein concentration ratio resulting in differential binding isotherms that can be adequately described by a two-site (panels a and b) or a one-site (panels c and d) binding model. Resulting binding parameters are summarized in Table 2. **e** NMR-titration of ^15^N-labeled TtSlyD_FL_ with the S2-P25A peptide at 25 °C. The relative change in chemical shift is plotted versus the total concentration of added peptide, with *red dots* corresponding to residues in the IF domain (S77, A78, V85, and V86) and *blue dots* to the FKBP domain (G46, F128, and A138). The error bars indicate the standard error. The *black solid curve* represents the theoretical binding isotherm calculated using K_D1_ = 0.13 μM and the *dashed curve* represents the theoretical binding isotherm calculated using K_D2_ = 7.0 μM, as obtained from the ITC measurements (Table 2). **f** Mean weighted chemical shift changes are plotted against residue number. Resonances completely broadened by peptide binding are indicated by *gray bars* covering the full vertical scale. The cut-off of 0.1 ppm is shown as a *red dotted line*. **g** Residues with a shift difference >0.1 ppm or completely broadened are highlighted in *red* on the structure of the TtSlyD_FL_:S2 complex [PDB: 4ODL], with the peptide shown in *blue*

To identify peptides that overcome the limitations of currently used substrate mimics and are suitable for structural studies, we used ITC to characterize the binding of several different peptides to TtSlyD, as exemplified in Fig. 1a–d. We mainly used 15-residue-long proline-containing segments from proteins that have previously been shown to bind to TtSlyD and/or other proteins from the SlyD family, namely RNase T1, which is a model protein for folding studies, and the ribosomal proteins S2 and S3 [17, 18, 24]; see Table 1 for the complete list of peptide sequences. Table 2 summarizes the results of the binding studies. Interestingly, peptides derived from S2 and S3 display a dual binding mode with both a high- and a low-affinity binding site (Fig. 1a, b). In order to identify the binding sites we next monitored peptide binding by heteronuclear NMR spectroscopy using a variant of the S2 peptide (S2-P25A). Chemical shift perturbations on full-length TtSlyD (henceforth abbreviated TtSlyD_FL_) upon addition of peptide clearly show binding to both the IF and FKBP domains (Fig. 1e–g). Quantitative analyses of chemical shift changes as a function of added peptide enabled us to assign the stronger binding event to the IF domain and the weaker one to the FKBP domain (Fig. 1e–g). The highest affinities were obtained for the S2 peptide with K_D1_ = 0.161 μM and K_D2_ = 2.97 μM, followed by the S3 peptide with K_D1_ = 0.869 μM and K_D2_ = 22.94 μM (Table 2). Addition of 5 % dimethyl sulfoxide (DMSO), which was required for dissolving some peptides at high concentration, histidine tag cleavage, or a change in temperature (20 °C versus 25 °C), had only minor effects on the affinity of the S2 peptide (Table 2). In general, peptide binding to TtSlyD_FL_ is driven by a favorable change in enthalpy (Table 2). A comparison of the thermodynamic fingerprints of the S2 and S3 peptides revealed that the slightly lower affinity of the S3 peptide to the IF domain is caused by a reduction in binding enthalpy (which is partly compensated by entropy), while the weaker binding of the S3 peptide to the FKBP domain is due to entropic effects (Table 2). For the T1 peptide, a single-site model was sufficient to describe binding to TtSlyD_FL_, and the affinity was found to be much lower (K_D_ = 158 μM) than for the S2 and S3 peptides, which is explained primarily by an increase in unfavorable binding entropy. Notably, the affinities observed for the S2 and S3 peptides are in the same range or higher than for binding of a refolding protein substrate at high salt concentration to *E. coli* SlyD (K_D_ = 0.4–2.2 μM) [19, 23], and significantly higher than the reported K_D_ of ~44 μM estimated for binding of the suc-ALPF-pNA peptide to *E. coli* SlyD at high salt concentration [23]. We therefore conclude that the S2 and S3 peptides can serve as improved substrate mimics for functional and structural studies of TtSlyD.

**Table 1:** Peptides used in this study

Name	Sequence	Charge	Source	
			UniProt	Position
S2	TRYWNPKMKPFIFGA	+3	P0A7V0	20–34
S2-minus1	GHQTRYWNPKMKPFI	+3 to +4		17–31
S2-minus2	VHFGHQTRYWNPKMK	+3 to +5		14–28
S2-minus3	KAGVHFGHQTRYWNP	+2 to +4		11–25
S2-plus1	WNPKMKPFIFGARNK	+4		23–37
S2-plus2	KMKPFIFGARNKVHI	+4 to +5		26–40
S2-long2	QTRYWNPKMKPFIFGAR	+4		19–35
S2-long4	HQTRYWNPKMKPFIFGARN	+4 to +5		18–36
S2-long6	GHQTRYWNPKMKPFIFGARNK	+5 to +6		17–37
S2-long8	FGHQTRYWNPKMKPFIFGARNKV	+5 to +6		16–38
S2-short2	RYWNPKMKPFIFG	+3		21–33
S2-short4	YWNPKMKPFIF	+2		22–32
S2-short6	WNPKMKPFI	+2		23–31
S2-short8	WNPKMKP	+2		23–29
S3	RLGIVKPWNSTWFAN	+2	P0A7V3	11–25
T1	VGSNSYPHKYNNYEG	0 to +1	P00651	59–73
SlpA linker	SGLVPRGS	+1	-	-

For peptides derived from the S2 protein, parts overlapping the original S2 peptide are underlined. The expected charges at neutral pH are listed. These were calculated using −1 for Glu and Asp, +1 for Lys and Arg, and 0 or +1 for His (for peptides with His residues, the charge is given as a range). In addition to the peptides listed here, we also used the following five mutants of the S2 peptide: P25A, P29E, P25A/P29E, P25N/P29N, and W23A

**Table 2:** Results from peptide binding studies

Construct	Peptide	N1	K_D_1 μM	ΔH1 (kcal/mol)	–T · ΔS1 (kcal/mol)	N2	K_D_2 μM	ΔH2 (kcal/mol)	–T · ΔS2 (kcal/mol)	Comments
TtSlyD_FL_	S2	0.92 ± 0.02	0.161 ± 0.020	–15.8 ± 0.11	6.6	1.00 ± 0.01	2.97 ± 0.22	–9.4 ± 0.36	1.8	25 °C
	S2	0.88 ± 0.02	0.128 ± 0.013	–16.6 ± 0.10	6.1	0.92 ± 0.01	2.23 ± 0.15	–10.5 ± 0.50	2.9	25 °C, tag-free
	S2	0.98 ± 0.01	0.150 ± 0.021	–14.8 ± 0.11	5.5	1.05 ± 0.01	3.16 ± 0.18	–8.9 ± 0.25	1.4	25 °C, 5 % DMSO
	S2	0.96 ± 0.03	0.113 ± 0.012	–10.0 ± 0.11	0.7	0.96 ± 0.02	2.93 ± 0.12	–4.9 ± 0.22	–2.5	20 °C
	S3	0.89 ± 0.07	0.869 ± 0.156	–9.1 ± 0.05	0.8	0.86 ± 0.04	22.94 ± 1.27	–9.8 ± 0.39	3.5	25 °C
	T1					1.13 ± 0.05	158 ± 19	–14.6 ± 0.80	9.5	25 °C
	SlpA linker					1.44 ± 0.08	133 ± 23	–0.4 ± 0.05	–4.9	25 °C
	S2-minus1	0.89 ± 0.02	0.667 ± 0.059	–16.6 ± 0.12	8.2	0.82 ± 0.02	7.25 ± 0.15	–11.1 ± 0.29	4.1	25 °C
	S2-minus2	0.96 ± 0.03	1.472 ± 0.133	–16.1 ± 0.21	8.1	0.99 ± 0.06	14.77 ± 1.09	–4.3 ± 0.92	–2.3	25 °C
	S2-minus3	0.94 ± 0.06	2.383 ± 0.321	–17.0 ± 0.73	9.4	1.17 ± 0.06	9.35 ± 0.49	–2.1 ± 1.41	–4.8	25 °C
	S2-plus1	1.06 ± 0.03	0.317 ± 0.037	–11.4 ± 0.13	2.3	0.94 ± 0.03	3.28 ± 0.08	–7.8 ± 0.23	0.4	25 °C
	S2-plus2	0.88 ± 0.02	0.183 ± 0.018	–13.1 ± 0.14	3.9	0.75 ± 0.04	5.68 ± 0.27	–8.4 ± 0.39	1.2	25 °C
	S2-long2	0.72 ± 0.01	0.077 ± 0.007	–10.8 ± 0.05	1.2	1.00 ± 0.01	6.62 ± 0.39	–5.5 ± 0.14	–1.4	20 °C
	S2-long4	0.74 ± 0.01	0.070 ± 0.004	–11.1 ± 0.04	1.5	0.87 ± 0.01	4.16 ± 0.20	–5.3 ± 0.10	–1.9	20 °C
	S2-long6	0.74 ± 0.01	0.035 ± 0.003	–10.5 ± 0.04	0.6	0.89 ± 0.01	4.52 ± 0.32	–4.7 ± 0.11	–2.5	20 °C
	S2-long8	0.95 ± 0.01	0.052 ± 0.006	–11.7 ± 0.03	1.9	0.71 ± 0.02	7.93 ± 0.46	–6.9 ± 0.22	–0.1	20 °C
	S2-short2	0.72 ± 0.03	0.232 ± 0.043	–9.7 ± 0.21	0.9	0.91 ± 0.02	3.16 ± 0.24	–5.5 ± 0.34	–1.9	20 °C
	S2-short4	0.73 ± 0.24	1.193 ± 0.666	–10.7 ± 2.33	2.7	0.87 ± 0.23	4.24 ± 0.50	–6.3 ± 3.02	–0.9	20 °C
	S2-short6					1.80 ± 0.01	21.93 ± 0.51	–5.4 ± 0.04	–0.8	20 °C
	S2-short8					1.29 ± 0.30	163.4 ± 26.4	–5.1 ± 1.54	–0.1	20 °C
	S2-P25A	0.92 ± 0.01	0.129 ± 0.028	–16.6 ± 0.16	7.2	1.02 ± 0.04	7.04 ± 1.27	–8.5 ± 0.82	1.5	25 °C
	S2-P29E	0.88 ± 0.02	0.513 ± 0.052	–15.8 ± 0.16	7.2	1.02 ± 0.04	5.68 ± 0.57	–2.7 ± 0.48	–4.5	25 °C
	S2-P25A/P29E	1.13 ± 0.03	1.447 ± 0.295	–14.1 ± 0.33	6.1	1.09 ± 0.24	44.05 ± 8.42	–5.9 ± 1.91	–0.1	25 °C, 5 % DMSO
	S2-P25N/P29N	0.93 ± 0.02	0.234 ± 0.043	–16.8 ± 0.12	7.8	1.18 ± 0.18	8.33 ± 1.54	–2.3 ± 0.33	–4.4	25 °C, 5 % DMSO
	SlpA linker P5T					1.76 ± 0.12	130 ± 20	–0.9 ± 0.10	–4.4	25 °C
	S2-W23A	0.76 ± 0.03	0.855 ± 0.332	–15.8 ± 0.24	7.5	0.91 ± 0.40	18.83 ± 6.2	–2.3 ± 0.97	–4.2	25 °C
TtSlyD_ΔIF_	S2					0.91 ± 0.01	14.43 ± 0.46	–10.0 ± 0.07	3.4	25 °C
	S2					0.88 ± 0.02	12.23 ± 0.24	–5.9 ± 0.11	0.4	20 °C
	S3					0.89 ± 0.07	34.25 ± 3.40	–10.5 ± 1.02	4.4	25 °C
	S2-P25A					0.91 ± 0.01	12.13 ± 0.76	–11.7 ± 0.24	5.0	25 °C
	S2-P29E					0.69 ± 0.03	13.81 ± 0.88	–10.8 ± 0.27	4.2	25 °C
	S2-P25A/P29E					0.84 ± 0.06	28.98 ± 4.62	–6.3 ± 0.58	0.1	25 °C, 5 % DMSO
	S2-W23A					0.77 ± 0.02	17.06 ± 1.13	–8.1 ± 0.25	1.6	25 °C

In most cases, the Isothermal titration calorimetry data clearly supported the presence of two binding sites. Where data could be described by only a single binding site, the results are given in the columns for binding site 2. Note that the binding experiments with the proline double mutant peptides were carried out in 5 % dimethyl sulfoxide (DMSO) due to solubility issues. However, this is not expected to appreciably affect the experiments, as the S2 peptide was found to bind with similar affinities in both the presence and absence of 5 % DMSO

In order to characterize the requirements for binding, we next analyzed binding of a series of variants of the S2 peptide (Table 1) to TtSlyD_FL_. To test the sequence dependency, we used a set of peptides corresponding to different segments of the S2 protein: three peptides covered sequences shifted N-terminally by three, six, or nine residues, compared to the original S2 peptide (S2-minus1, S2-minus2, and S2-minus3), and two peptides were shifted C-terminally by three or six residues (S2-plus1 and S2-plus2). These peptides all retained the capacity for binding to both sites, though the affinities were most often reduced when compared to the original S2 peptide (Table 2). In most cases, enthalpy losses were to some extent compensated by reduced entropy penalties and, in some cases, even gains in entropy (Table 2). To study the length dependency, we used variants of the S2 peptide extended or truncated with up to eight residues. Extension of the peptide had minor to moderate effects on the affinity for the IF domain (up to threefold higher) and the FKBP domain (up to threefold lower) (Fig. 1b and Table 2). Removing one or two residues from each side (S2-short2 and S2-short4) reduced the affinity for the IF domain about twofold and sevenfold, respectively, while the affinities for the FKBP domain remained relatively unperturbed. After removing an additional two to four residues (S2-short6 and S2-short8), the affinities were substantially reduced, and it was no longer possible to resolve the separate binding events (Fig. 1c and Table 2). The stoichiometries were, however, significantly higher than 1 after fitting to a single binding site model, indicating that these peptides may still engage both binding sites (Table 2). We also measured binding of an eight-residue-long peptide representing the plasmid-derived linker sequence bound to the IF domain in the crystal structure of SlpA (SlpA linker; [18]), which was found to bind with an affinity similar to that of the seven-residue-long S2-short8 peptide. We conclude that the sequence requirements for peptide binding to TtSlyD_FL_ are rather lax, which is in part because the enthalpic losses incurred by apparent sequence mismatching are readily compensated by gains in entropy, and that the affinity of binding to both domains is sensitive to the length of the peptide (Table 2).

Next we tested the contribution of proline residues to the binding events. There are two proline residues in the S2 peptide, P25 and P29. Neither single mutations (P25A or P29E), nor double mutations (P25A/P29E or P25N/P29N) abrogated binding to any of the two domains, though the affinities were moderately reduced in most cases, and strongly so for the P25A/P29E double mutant (Table 2). Furthermore, mutating the single proline residue in the SlpA linker peptide to threonine did not appreciably alter the affinity of this peptide for TtSlyD_FL_ (Table 2). We therefore conclude that binding of substrates to TtSlyD_FL_ does not strictly require the presence of proline residues. Next, we tested binding of an S2 mutant peptide where the sole tryptophan residue was replaced by alanine (S2-W23A). This peptide retained the ability to bind to both domains of TtSlyD_FL_, albeit with moderately reduced affinities (Table 2). The results using point-mutated peptide variants thus further underline the lax sequence specificity of TtSlyD. The thermodynamic parameters of binding to the IF domain are similar for the wild-type and point-mutated peptides, but the enthalpies of binding to the FKBP domain are significantly reduced for the P29E, P25A/P29E, P25N/P29N, and W23A variants, which is again partly compensated for by gains in entropy (Table 2).

Finally, we also analyzed binding of the S2, S3, and mutated variants of the S2 peptide to a chimeric TtSlyD construct, in which the IF domain is replaced by the flap loop from human FKBP12 (henceforth abbreviated TtSlyD_ΔIF_). As expected, only one binding event was observed in this case (Fig. 1d and Table 2). Furthermore, the affinities for the FKBP domain were found to be 1.5–4.8-times lower in the absence of the IF domain (except for the S2-W23A and S2-P25A/P29E peptides), which is primarily due to a more unfavorable binding entropy (Table 2).

### Overall structure of TtSlyD in complex with peptides and FK506

**Fig. 2:**
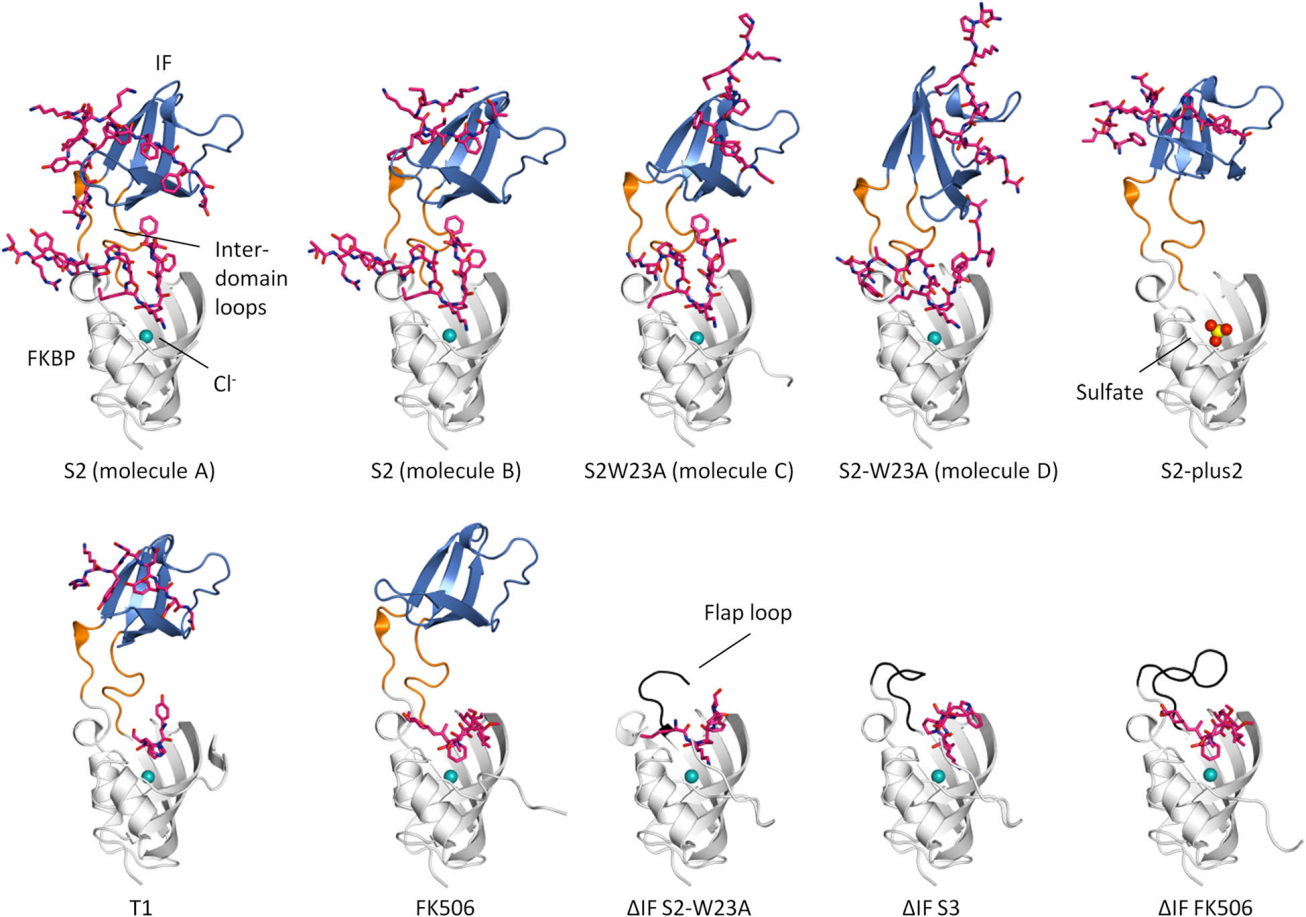
Overview of structures. The structures of all five full-length TtSlyD (TtSlyD_FL_) and three TtSlyD constructs with the insert-in-flap (IF) domain replaced by the flap loop from human FKBP12 (TtSlyD_ΔIF_) are shown in ribbon representation with the FK506-binding protein (FKBP) domain in *white*, IF domain in *blue*, inter-domain loops in *orange* (TtSlyD_FL_), and flap loop in *black* (TtSlyD_ΔIF_). *Spheres* designate bound anions (chloride is *turquoise* and sulfate is *yellow/red*), and *pink sticks* represent the bound peptides and FK506. All structures are shown in the same orientation and are labeled according to which substrate is bound. Note that the TtSlyD_FL_:S2 and TtSlyD_FL_:S2W23A structures display different peptide binding modes for the different TtSlyD_FL_ copies in their asymmetric units. TtSlyD_FL_:S2 thus presents two different binding modes at the IF domains of TtSlyD_FL_ molecules A and B (both are shown), while TtSlyD_FL_:S2W23A displays two different binding modes at the FKBP domain of molecules A and C contra molecules B and D (shown for molecules C and D). Additional file 2 shows the binding site for the chloride ion in detail, and Additional file 3 shows a metal binding site that was omitted from the main figure for clarity

Three structures of TtSlyD_FL_ were known prior to this study (maximum resolution of 2.7–2.4 Å): two apo structures [PDB: 3CGM and 3CGN] and one with the modified tetrapeptide suc-ALPF-pNA bound to the FKBP domain [PDB: 3LUO] [17]. However, no structures had been determined for TtSlyD, or any other member of the SlyD family, in complex with longer, unmodified peptides or with inhibitors. We therefore co-crystallized the full-length and ΔIF constructs of TtSlyD with several of the peptides identified as substrates in the binding studies, namely the S2, S3, T1, S2-plus2, and S2-W23A peptides. In addition, we carried out co-crystallization with the FKBP inhibitor FK506. For phasing, we used both molecular replacement and single-wavelength anomalous diffraction (SAD), as detailed in the “Methods.” In total, we obtained five structures of TtSlyD_FL_ and three of TtSlyD_ΔIF_ at a maximum resolution of up to 1.4 Å (Fig. 2 and Table 3). Furthermore, there is more than one molecule of TtSlyD in the asymmetric unit in several cases (Table 3), which for TtSlyD_FL_:S2 and TtSlyD_FL_:S2-W23A display substantial differences in substrate binding (Fig. 2). In most of the TtSlyD_FL_ complexes, a peptide is bound to each of the IF and FKBP domains (Fig. 2). Notably, the positions of the binding sites in the crystal structures are the same as in solution, as confirmed by mapping of the chemical shift perturbations (Fig. 1g).

**Table 3:** Crystallographic data processing and refinement statistics

	TtSlyD_FL_:T1	TtSlyD_FL_:S2	TtSlyD_FL_:S2-W23A	TtSlyD_FL_:S2-plus	TtSlyD_FL_:FK506	TtSlyD_∆IF_:S2-W23A	TtSlyD_∆IF_:S3	TtSlyD_∆IF_:FK506
Data collection								
Beamline	Diamond I24	Diamond I03	Diamond I02	Diamond I04-1	Diamond I24	Diamond I24	SOLEIL Proxima 1	SOLEIL Proxima 1
Wavelength (Å)	0.9191	0.9795	0.9199	0.9200	1.0000	1.4000	1.0332	0.9801
Space group	C2	P6_2_22	C2	P2_1_	P1	P4_1_32	P4_1_32	P4_3_2_1_2
Cell dimensions								
*a*, *b*, *c* (Å)	87.50 52.99 48.91	110.88 110.88 182.31	184.12 41.20 131.52	39.83 40.55 53.22	48.71 50.21 57.67	93.69 93.69 93.69	93.56 93.56 93.56	72.50 72.50 178.96
α, β, γ (°)	90 111.18 90	90 90 120	90 123.13 90	90 91.24 90	85.74 68.92 80.12	90 90 90	90 90 90	90 90 90
Resolution (Å)	29.10–1.40 (1.44–1.40)	48.01–2.92 (2.99–2.92)	29.11–1.75 (1.80–1.75)	28.41–1.60 (1.64–1.60)	27.81–1.60 (1.64–1.60)	29.63–1.75 (1.79–1.75)	38.20–2.00 (2.05–2.00)	44.48–1.93 (1.98–1.93)
*R*_*sym*_	0.023 (0.714)	0.047 (0.679)	0.047 (0.766)	0.097 (0.772)	0.052 (0.568)	0.044 (0.872)	0.106 (0.774)	0.048 (0.777)
*I/σI*	23.03 (2.02)	30.68 (3.27)	18.31 (2.42)	12.74 (2.29)	14.46 (2.47)	35.85 (3.60)	23.33 (4.38)	26.27 (2.75)
Completeness (%)	97.6 (93.7)	99.1 (99.8)	98.9 (97.6)	99.3 (97.7)	95.3 (87.4)	100 (99.9)	100 (100)	99.9 (99.5)
Total number of reflections	134,551 (9,116)	126,159 (9,085)	307,806 (22,441)	151,369 (9,923)	219,115 (13,672)	475,595 (32,633)	417,094 (29,168)	523,651 (36,435)
Multiplicity	3.3 (3.2)	8.4 (8.5)	3.7 (3.7)	6.7 (6.1)	3.5 (3.2)	17.6 (16.4)	23.3 (22.1)	7.6 (7.2)
Wilson *B*-factor (Å)	23.11	84.82	26.98	15.67	20.93	29.88	26.17	32.03
Refinement								
*R*_work_ / *R*_free_	0.139 / 0.167	0.210 / 0.224	0.176 / 0.208	0.177 / 0.198	0.180 / 0.202	0.184 / 0.202	0.199 / 0.215	0.156 / 0.168
Number of TtSlyD/ASU	1	2	4	1	3	1	1	2
Number of atoms								
Protein	1,229	2,314	4,708	1,179	3,558	814	843	1,650
Peptides/FK506	102	469	705	105	171	43	49	114
Water	195	6	606	228	630	100	107	316
Other solvent	1	2	20	30	27	6	6	53
*B*-factors								
Protein	30.1	71.2	32.4	15.6	29.4	41.1	29.1	31.0
Peptides/FK506	36.0	88.9	45.7	33.7	28.6	78.7	45.5	32.2
Solvent	45.4	41.6	42.3	34.6	39.1	46.0	35.8	50.2
R.m.s. deviations								
Bond lengths (Å)	0.005	0.003	0.006	0.017	0.004	0.016	0.009	0.014
Angles (°)	1.027	0.759	1.094	1.177	0.947	1.258	0.839	1.100
Ramachandran								
Favored (%)	99.4	95.0	98.5	98.8	98.7	100	97.2	98.1
Outliers (%)	0.0	0.3	0.0	0.0	0.0	0.0	0.0	0.0
Clash score	1.9	2.0	2.2	4.7	4.3	2.4	2.9	4.7
PDB accession	4ODK	4ODL	4ODM	4ODN	4ODO	4ODP	4ODQ	4ODR

All three TtSlyD_∆IF_ data sets were processed anomalously, while the full-length TtSlyD data sets were not. Numbers in parentheses refer to statistics for the outer shell. The Ramachandran statistics and clash scores were determined using the MolProbity validation tool

**Fig. 3:**
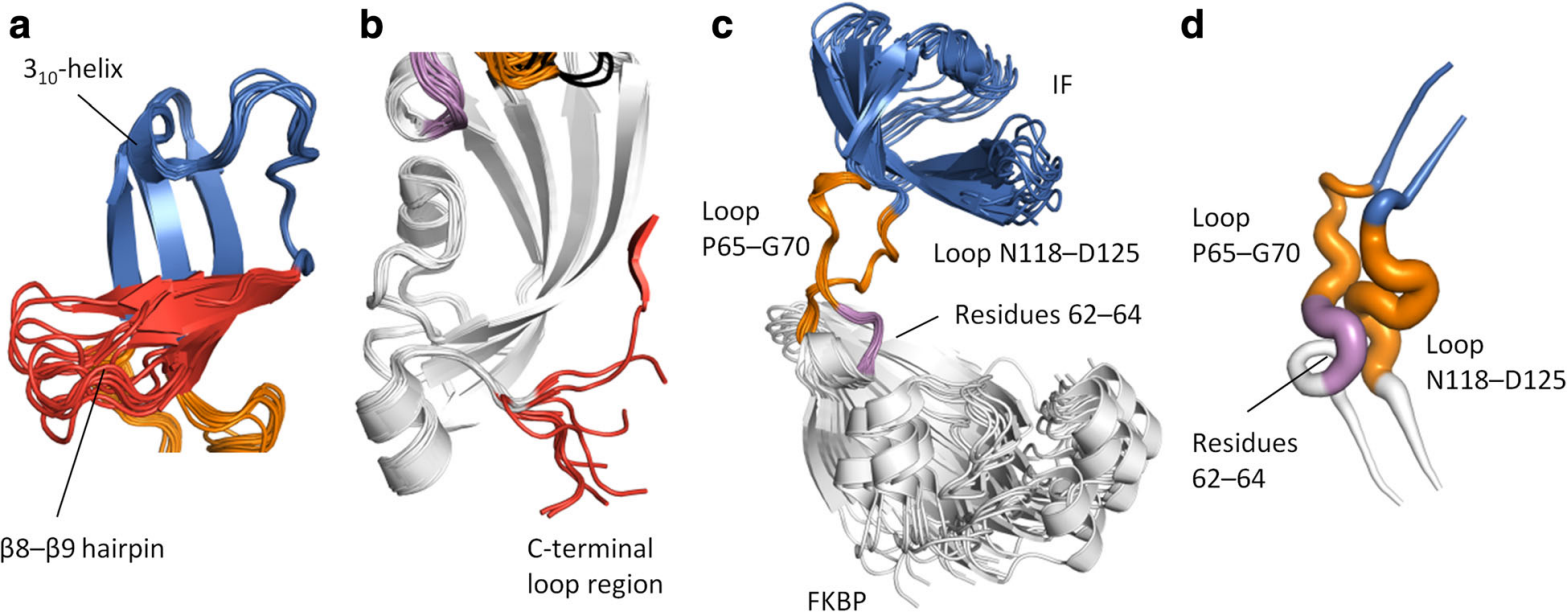
Structural flexibility. **a** Superimposition of the insert-in-flap (IF) domain for all molecules of the five full-length TtSlyD (TtSlyD_FL_) structures. Colored as in Fig. 2 except that the structurally most variable region is *red* (this region corresponds to the β8–β9 hairpin). **b** Superimposition of the FK506-binding protein (FKBP) domain for all eight TtSlyD_FL_ structures and TtSlyD constructs with the insert-in-flap (IF) domain replaced by the flap loop from human FKBP12 (TtSlyD_ΔIF_). Colored as in Fig. 2, except that residues 62–64, which can be considered as part of both the FKBP domain and the connector loops, are *violet*, and that the structurally most variable region is *red* (this region corresponds to the C-terminal tail). **c** All TtSlyD_FL_ molecules of the five TtSlyD_FL_ structures were superimposed on the inter-domain loops (except TtSlyD_FL_:FK506 molecule B, where the loops were uncharacteristically poorly defined in the electron density map). **d** A DynDom computational analysis was carried out to identify putative hinge regions between the domains based on pair-wise superimpositions of all possible combinations of TtSlyD_FL_ molecules. The results are displayed in a “putty” representation: the more commonly a given residue was found to be part of a hinge region, the thicker the putty trace. For loop_65–70_, the thick part clearly converges around residues 62–64 at the N-terminal junction, while it is more diffusely distributed for loop_118–125_. Additional file 1 shows ^1^H–^13^C transverse relaxation optimized spectroscopy hetero single quantum coherence (TROSY-HSQC) spectra of the Fδ,ε/Yδ and Yε region data that corroborate the results from the DynDom analysis

Apart from the differences in substrate binding, the IF and FKBP domains show only small structural variations except in the β8–β9 hairpin (residues 90–109) of the IF domain (Fig. 3a) and the C-terminal tail of the FKBP domain (Fig. 3b), which is in line with previous results on TtSlyD and other members of the SlyD family [17–19]. The IF and FKBP domains are connected via loop_65−70_ and loop_118−125_, which adopt the same conformations as previously described for other members of the SlyD family [18]. However, in spite of the loops being structurally well defined, some degree of bending must take place in these regions, because the relative orientation of the two domains is quite variable (Fig. 3c). A computational analysis suggests that there is a key pivot point in the hinge region comprising residues 62–64 (Fig. 3d), which can be considered part of both the FKBP domain and the inter-domain connectors, and which is also conserved in many other FKBPs where it forms the N-terminal base of the flap loop [15]. A high mobility of the Y63 side chain is furthermore confirmed by aromatic ^1^H–^13^C NMR spectra of apo TtSlyD_FL_, where Y63 is the only aromatic residue completely broadened beyond detection due to conformational exchange dynamics affecting both the δ and ε positions (Additional file 1). As discussed further below, this variability is intimately connected with interactions between the side chain of Y63 and the peptides bound to the FKBP domain. The FKBP12 flap loop inserted into TtSlyD_ΔIF_ in place of the IF domain adopts essentially the same conformation as in full-length FKBP12 in the case of TtSlyD_ΔIF_:FK506, while it is partially disordered in TtSlyD_ΔIF_:S2-W23A and TtSlyD_ΔIF_:S3 (Fig. 2).

A previously unrecognized chloride anion is bound near the binding site of the FKBP domain in all of the new structures, except TtSlyD_FL_:S2-plus2 where it is replaced by sulfate (Fig. 2 and Additional file 2), and a metal ion is bound in a previously identified binding site near the C-terminus [17] in the TtSlyD_ΔIF_ structures, but not the TtSlyD_FL_ structures (Additional file 3). The role of the chloride ion is unknown, whereas the metal binding site could be important for facilitating metal insertion and folding of metalloproteins [17].

### Peptide binding to the IF domain

**Fig. 4:**
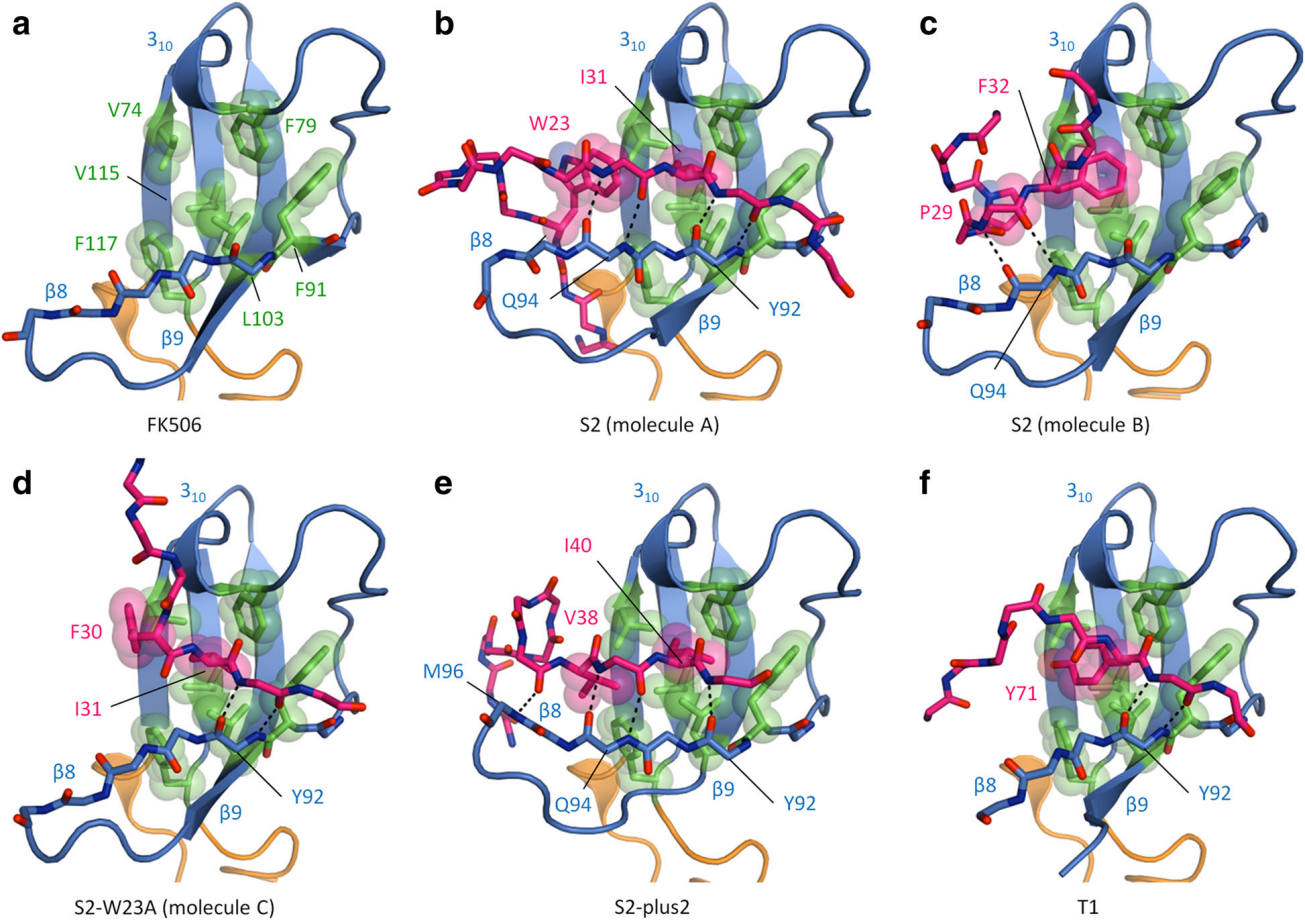
Binding of peptides to the insert-in-flap (IF) domain. **a** The substrate-free IF domain of TtSlyD_FL_:FK506 (molecule B) is depicted as in Fig. 2, except that the backbone of β8 is shown in *sticks*, and the side chains of residues forming a hydrophobic binding groove are shown in *green sticks* and *semi-transparent spheres*. **b** Binding of the S2 peptide to the IF domain of molecule A in the TtSlyD_FL_:S2 structure. The backbone of the peptide is shown in *pink sticks*, and the hydrophobic side chains that are sequestered in the hydrophobic binding groove are shown in *sticks* and *semi-transparent spheres. Dashes* indicate main chain hydrogen bonds between the peptide and β8. **c** Peptide binding in TtSlyD_FL_:S2 (molecule B). **d** Peptide binding in TtSlyD_FL_:S2-W23A (molecule C). **e** Peptide binding in TtSlyD_FL:_S2-plus2. **f** Peptide binding in TtSlyD_FL_:T1. Additional file 4 shows the electron density map for the S2 peptides bound to the IF domain, Additional file 5 shows an analysis of the structural changes in the IF domain induced by substrate binding, and Additional file 6 shows the peripheral substrate:IF domain interactions

A peptide is bound to the IF domain in four structures: TtSlyD_FL_:S2, TtSlyD_FL_:S2-W23A, TtSlyD_FL_:S2-plus2, and TtSlyD_FL_:T1 (Figs 2 and 4). Indeed, the only structure with a substrate-free IF domain is TtSlyD_FL_:FK506 (Fig. 4a), which was crystallized in the absence of a peptide. The binding site consists of a highly hydrophobic groove comprising V74, F79, F91, L103, V115, and F117, as well as the edge of the β8–β9 hairpin (Fig. 4a). The binding mode is highly variable among the structures. Moreover, in the case of TtSlyD_FL_:S2, there are two TtSlyD_FL_ molecules in the asymmetric unit (molecules A and B), which bind the peptides in very different ways (Fig. 4b, c). The resolution of this structure is, however, rather low (2.9 Å), and although the electron density map clearly shows that the peptide binds differently to the IF domains of molecules A and B, the side chains are poorly defined for the peptide bound to molecule B (Additional file 4). The S2 peptide inserts W23_S2_ and I31_S2_ into the binding groove of molecule A (Fig. 4b), whereas it appears to insert P29_S2_ and F32_S2_ in molecule B (Fig. 4c). We therefore expected that a peptide where W23_S2_ is mutated to alanine would bind similarly to the S2 peptide bound to molecule B in the TtSlyD_FL_:S2 structure. Surprisingly, the structure of TtSlyD_FL_:S2-W23A revealed instead a third binding mode where F30_S2_ and I31_S2_ are inserted into the binding groove (Fig. 4d). Furthermore, although the S2-plus2 peptide encompasses the same residues that form most of the intermolecular contacts in both the TtSlyD_FL_:S2 and TtSlyD_FL_:S2-W23A structures, including W23_S2_, P29_S2_, I31_S2_, and F32_S2_ (Table 1), it was found to bind in yet a fourth mode with V38_S2_ and I40_S2_ inserted into the binding groove (Fig. 4e). Interestingly, although the binding mode is variable, three aspects are shared for all structures: (i) the peptides bind to the β8–β9 hairpin through β-strand augmentation with two to four hydrogen bonds (Fig. 4b–f), (ii) the peptides generally insert two hydrophobic side chains into the binding groove as detailed above (Fig. 4b–e), and (iii) peptide binding does not perturb the hydrophobic groove (Additional file 5). The only exception to rule (ii) is the T1 peptide, which inserts only one hydrophobic residue (Y71_T1_; Fig. 4f), but this peptide also binds with much lower affinity than the S2, S2-W23A, and S2-plus2 peptides (Table 2). In addition to the shared core interactions, a number of highly variable peripheral interactions are also formed. These mainly include van der Waal interactions (in particular with the β8–β9 hairpin), but also some hydrogen bonds, as well as a single salt bridge in the case of TtSlyD_FL_:S2-plus2 (for more details, see Additional file 6). The fact that rather extensive interactions are formed between the IF domain and the peptides is also reflected in the favorable binding enthalpy measured by ITC. Interestingly, the proline residues of the peptides bound to the IF domains are all in the *trans* conformer—with the possible exception of the S2 peptide bound to molecule B in the TtSlyD_FL_:S2 structure, where P29_S2_ could be in *cis* configuration (Fig. 4c and Additional file 4)—and in most cases do not form direct interactions with the IF domain. This is in good agreement with the binding studies, which showed that the IF domain is capable of binding peptides both with and without proline residues with high affinity.

### The binding site of the FKBP domain

**Fig. 5:**
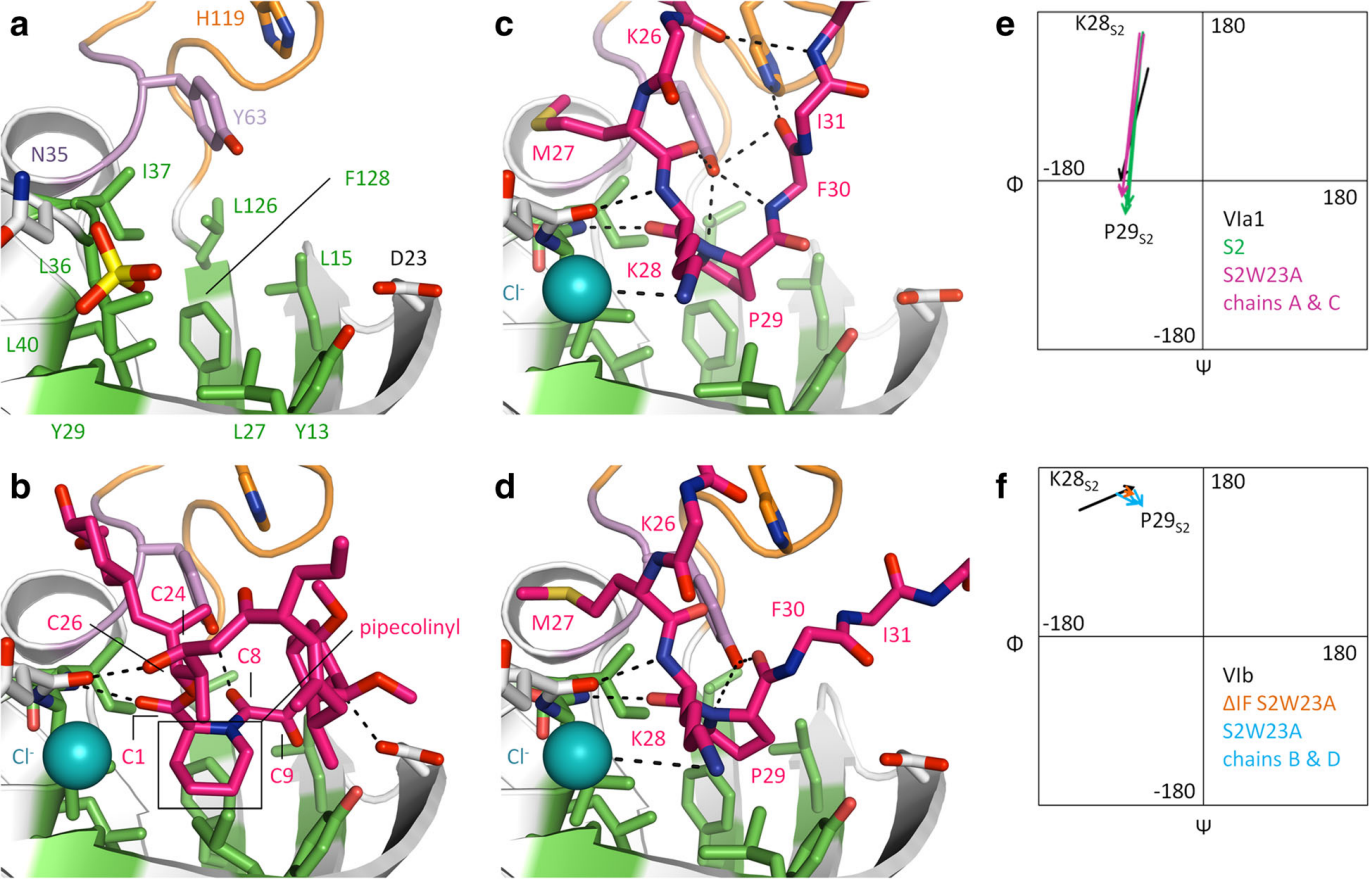
Binding of peptides and FK506 to the FK506-binding protein (FKBP) domain. **a** The substrate-free FKBP domain of TtSlyD_FL_:S2-plus2. The color scheme is the same as used in Fig. 2, except that residues 62–64 are *violet*, and the residues forming the hydrophobic binding pocket are *green*. Residues involved in binding are shown in *sticks*. **b** Binding of FK506 to full-length TtSlyD (TtSlyD_FL_). *Dashes* indicate distances up to 3.5 Å between conventional hydrogen bond donors and acceptors (weaker bonds such as CH–O and CH–π are omitted). The pipecolinyl ring and selected atoms of FK506 are labeled. **c** Binding of the S2-W23A peptide to molecule A in TtSlyD_FL_:S2-W23A. A similar binding mode is also seen for molecule C and TtSlyD_FL_:S2. For clarity, side chains are only shown for residues 27–29 of the peptide. The residues of the peptide are labeled. **d** Binding of the S2-W23A peptide to molecule D in TtSlyD_FL_:S2-W23A. A similar binding mode is also seen for molecule B and TtSlyD_ΔIF_:S2-W23A. **e** Ramachandran plots for residues K28_S2_ and P29_S2_ of TtSlyD_FL_:S2 as well as TtSlyD_FL_:S2-W23A molecules A and C compared to the standard values for i + 1 and i + 2 residues of a type VIa1 β-turn. There is a clear match. **f** Similar Ramachandran plots for residues K28_S2_ and P29_S2_ of TtSlyD_FL_:S2-W23A molecules B and D as well as TtSlyD_ΔIF_:S2W23-A compared to the standard values of a type VIb β-turn. There is a partial match, but the phi angle of K28_S2_ (position i + 1) is off by 40–60°. Additional file 7 shows an analysis of the structural changes in the FKBP domain induced by substrate binding, Additional file 8 illustrates the non-canonical binding modes of the T1 and S3 peptides, and Additional file 9 shows the peripheral substrate:FKBP domain interactions

We determined several crystal structures of TtSlyD_FL_ and TtSlyD_ΔIF_ with either a long peptide or FK506 bound to the FKBP domain, namely TtSlyD_FL_:S2, TtSlyD_FL_:S2-W23A, TtSlyD_FL_:T1, TtSlyD_FL_:FK506, TtSlyD_ΔIF_:S2-W23A, TtSlyD_ΔIF_:S3, and TtSlyD_ΔIF_:FK506, whereas the TtSlyD_FL_:S2-plus2 structure displayed a substrate-free FKBP domain (Fig. 2). To the best of our knowledge, these represent the first structures of an FKBP domain in complex with long, unmodified peptides, and the first structures of a member of the SlyD family in complex with a macrolide inhibitor. Both FK506 and the peptides bind in the hydrophobic pocket, which is composed of numerous hydrophobic and aromatic residues, including Y13, L15, L27, Y29, L36, I37, L40, L126, and F128, and is flanked by D23, Y63, and H119 as well as the bound anion (Fig. 5a). Binding of FK506 or peptides strongly affect the position of Y63, as further described below, and the loop encompassing L36 and I37 also moves slightly, but otherwise the binding site remains relatively unperturbed (Additional file 7).

### Binding of FK506 to the FKBP domain

FK506 binds in a similar way to all three copies of TtSlyD in the asymmetric unit of TtSlyD_FL_:FK506 and in both copies in TtSlyD_ΔIF_:FK506 (Fig. 5b), but exhibits some structural variation in parts of the molecule that are more distal to the binding pocket. The binding mode is similar to that observed in the FK506–FKBP12 complex [25]. Specifically, the pipecolinyl ring, which mimics a proline side chain, is inserted into the center of the hydrophobic pocket, and four hydrogen bonds are formed: two to the backbone of N35 and I37 (V55 and I56 in FKBP12), and one each to the side chains of D23 and Y63 (D37 and Y82 in FKBP12) (Fig. 5b). Apart from these conventional hydrogen bonds, a number of weaker CH–O hydrogen bonds are also present. Most noteworthy are three potential interactions between the C9 carbonyl oxygen of FK506 and CH groups of Y13, L15, and F128, which are reminiscent of three putative CH–O interactions observed between FK506 and FKBP12 residues Y26, F36, and F99 [25]. Indeed, Y26 and F99 are equivalent to TtSlyD residues Y13 and F128, respectively.

### Peptide binding to the FKBP domain

The S2 and S2-W23A peptides both bind by inserting the side chain of P29_S2_ in *cis* form into the center of the binding pocket (Fig. 5c, d). The binding mode is the same for both TtSlyD_FL_ molecules in the TtSlyD_FL_:S2 structure (Fig. 5c), whereas the S2-W23A peptide binds in two different ways to the four molecules in the TtSlyD_FL_:S2-W23A structure: one similar to the S2 peptide (molecules A and C), and one in a different but partially overlapping fashion (molecules B and D; Fig. 5d). This latter binding mode was also observed in the TtSlyD_ΔIF_:S2-W23A structure. The TtSlyD_FL_:T1 and TtSlyD_ΔIF_:S3 structures both show alternative binding modes. The T1 peptide adopts a reverse orientation as compared to the S2 and S2-W23A peptides, and forms only few interactions with the FKBP domain (Additional file 8). The S3 peptide binds to TtSlyD_ΔIF_ by inserting a valine instead of a proline residue into the binding pocket (Additional file 8), which supports the conclusion that in vitro binding to the FKBP domain does not strictly require the presence of proline residues. The non-canonical binding modes agree well with the weaker affinities of these peptides, but it is unclear if they mimic any physiologically relevant interactions.

The two different binding modes observed for the S2 and S2-W23A peptides are characterized by different β-turn conformations. β-turns consist by definition of four residues with a distance between the Cα atoms of residues i and i + 3 of 7 Å or less, and are divided into nine types according to the phi and psi torsion angles of residues i + 1 and i + 2, with the additional requirement for types VIa1, VIa2, and VIb that i + 2 must be a *cis*-proline [5]. We found that the two binding modes observed for the peptides bound to both TtSlyD_FL_ molecules in the TtSlyD_FL_:S2 crystal, and for the peptide bound to TtSlyD molecules A or C in the TtSlyD_FL_:S2-W23A crystal (Fig. 5c), conform to a type VIa1 β-turn with *cis*-P29_S2_ in position i + 2 (Fig. 5e), whereas the conformation of the peptide bound to TtSlyD molecules B or D in the TtSlyD_FL_:S2-W23A crystal, as well as in TtSlyD_ΔIF_:S2-W23A (Fig. 5d), conforms to a type VIb-like β-turn with a distorted i + 1 phi angle (Fig. 5f).

A number of interactions are shared between the VIa1 and VIb-like binding modes (Fig. 5c, d): (i) two β-strand type interactions are formed between K28_S2_ and N35 and I37 of TtSlyD, (ii) the backbone nitrogen of P29_S2_ is within potential hydrogen-bonding distance (3.5 Å) of the hydroxyl group of Y63 from TtSlyD, (iii) the side chains of M27_S2_, K28_S2_, and P29_S2_ interact in a similar way with the binding pocket via van der Waal and hydrophobic interactions, and (iv) K28_S2_ interacts electrostatically with the bound chloride ion. Note that although K28_S2_ is well accommodated, the binding pocket clearly has room for larger side chains. Indeed, when the activity of SlyD from *E. coli* was screened with an Ala-X-Pro-Phe tetrapeptide with each of the 20 proteinogenic residues in the “X” position, aromatic residues were found to yield the highest k_cat_/K_M_ values, while lysine was in the middle range [26]. Notwithstanding the listed similarities, there are, however, a number of differences between the two binding modes. Most notably, Y63 forms different interactions in the two forms apart from the shared potential hydrogen bond with the backbone nitrogen of P29_S2_: in the VIa1 form, the hydroxyl group of the Y63 side chain is within hydrogen-bonding distance of the backbone nitrogen of F30_S2_ and the carbonyls of M27_S2_ and I31_S2_ (Fig. 5c), while in the VIb-like form, it is instead within hydrogen-bonding distance of the backbone carbonyl of P29_S2_ (Fig. 5d). In addition, a hydrogen bond is formed between F30_S2_ and H119 for the VIb-like form, but not the VIa1 form, and several differences are also observed in peripheral interactions (Additional file 9). Notably, K26_S2_, M27_S2_, and K28_S2_ adopt almost the same conformations in both binding modes. The differences in binding mode of the VIa1 and VIb-like forms thus lie mainly in the residues that are found in the C-terminal direction from P29_S2_ (Fig. 5c, d).

### Comparison of the binding modes of FK506 and the peptides

**Fig. 6:**
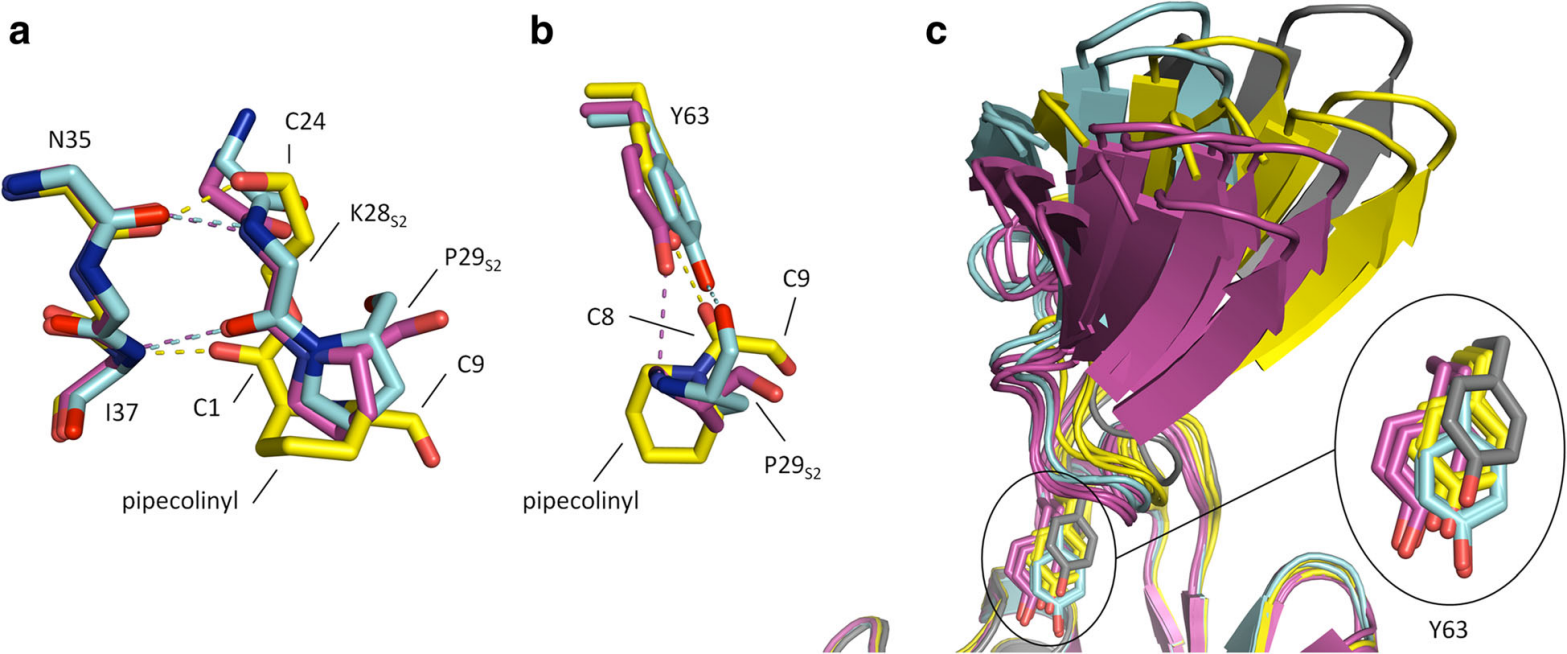
Comparison of binding modes for the FK506-binding protein (FKBP) domain. **a** Superimposition of the FKBP domains of TtSlyD_FL_:S2-W23A molecule B (*purple*), TtSlyD_FL_:S2-W23A molecules D (*light blue*), and TtSlyD_FL_:FK506 (*yellow*), showing the variation in interactions with the backbone of N35–I37. Selected amino acid residues and FK506 atoms are labeled. **b** Same overlay but showing instead the interaction with Y63. **c** Superimposition of the FKBP domains of all full-length TtSlyD (TtSlyD_FL_) molecules in the TtSlyD_FL_:S2-W23A, TtSlyD_FL_:S2, TtSlyD_FL_:FK506, and TtSlyD_FL_:S2-plus2 structures, showing how the orientation of the insert-in-flap (IF) domain varies with the position of Y63, and thus with which substrate is bound. TtSlyD_FL_ molecules binding the peptides in the type VIa1 or VIb-like modes are *purple* and *light blue*, respectively, TtSlyD_FL_:FK505 is *yellow*, and TtSlyD_FL_:S2-plus2 (apo form) is *gray*

A comparison of the FK506 and peptide binding modes reveals that the interactions between FK506 and the backbone atoms of N35 and I37, which are mediated by the O2 carbonyl oxygen atom at C1 and the O10 hydroxyl group at C24, respectively, overlap with the two β-strand type interactions formed by K28_S2_ in the S2 and S2-W23A peptides (Fig. 6a). It furthermore shows that the pipecolinyl ring partially overlaps with the side chain of *cis*-P29_S2_ in both peptide binding modes, but is in a roughly orthogonal orientation relative to these (Fig. 6b), which enables it to reach considerably deeper into the pocket. Moreover, there is a partial overlap between the large appendage at the C26 atom of FK506 and the side chain of M27_S2_ (Fig. 5b–d). The pipecolinyl ring is flanked by a dicarbonyl moiety encompassing both an amide carbonyl at C8 and an α-keto carbonyl at C9 (Fig. 6b), which are both candidates for mimicking the carbonyl group of a bound proline residue [25, 27, 28]. The carbonyl at C8 is in the *trans* form, as was also observed in the FKBP12:FK506 complex [25]. Yet, it still hydrogen bonds with Y63 similarly to the *cis*-P29_S2_ residue of the type VIb-like peptide, though at a different angle (Fig. 6b). This is made possible through the rotation of the pipecolinyl ring described above combined with a difference in the proline phi and psi angles relative to the equivalent angles in FK506 and a slight change in the position of Y63 (Fig. 6b). The carbonyl group at C9 is almost orthogonal to the carbonyl at C8 (Fig. 6b), which has been suggested to enable FK506 to mimic the twisted transition state [28]. It does not form conventional hydrogen bonds, but points directly into a sub-pocket of the binding site formed by Y13, L15, and F128, with which it forms CH–O hydrogen bonds, as described above. As hinted above, the position of Y63 differs depending on which substrate is bound. Indeed, it does not only differ between FK506 and peptides, but also between peptides adopting different binding modes (Fig. 6b). This may suggest that the flexibility of the hinge region encompassing Y63 is important for enabling the FKBP domain to adapt to different substrates. Furthermore, the different positions of Y63 also translate into different positions of the IF and FKBP domains relative to each other (Fig. 6c), which may be relevant in relation to the reported cross talk between them [23, 29, 30].

### Enzymatic activity

**Fig. 7:**
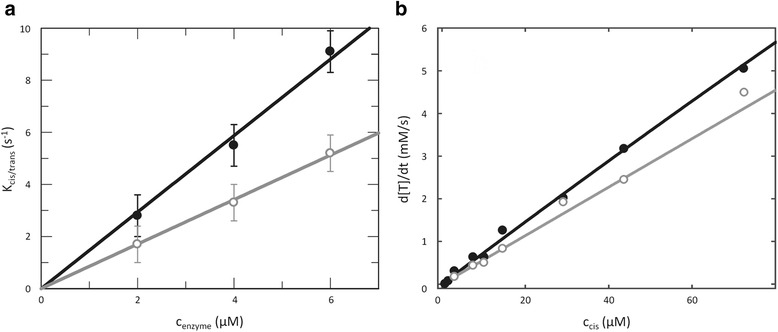
Michaelis-Menten analysis of TtSlyD at 25 °C. The data for full-length TtSlyD (TtSlyD_FL_ are shown as *black filled circles*, and the data for TtSlyD constructs with the insert-in-flap (IF) domain replaced by the flap loop from human FKBP12 (TtSlyD_ΔIF_) are shown as *gray open circles*. **a** Determination of k_cat_/K_M_ using the suc-ALPF-pNA tetrapeptide as substrate. The enzyme concentrations were varied, while 500 μM suc-ALPF-pNA (<< K_M_) was used in all experiments. We determined k_cat_/K_M_ to be 1.47 ± 0.05 • 10^6^ M^−1^s^−1^ for TtSlyD_FL_ and 0.85 ± 0.01 • 10^6^ M^−1^s^−1^ for TtSlyD_ΔIF_, using linear regression analysis. **b** Full Michaelis-Menten analysis using the S2-P25A peptide as substrate. A quantity of 0.2 μM of enzyme was used in all experiments, while the peptide concentration was varied. By using the previously determined *cis* content of 14.5 %, these concentrations were transformed into concentrations of S2-P25A *cis*. Michaelis-Menten fitting of TtSlyD_FL_ resulted in a k_cat_/K_M_ of 3.68 ± 0.04 • 10^8^ M^−1^s^−1^ with k_cat_ = 740,000 ± 140,000 s^−1^ and K_M_ = 2000 ± 410 μM. Because less curvature and precision was obtained for TtSlyD_ΔIF_, only the k_cat_/K_M_ of 2.84 ± 0.01 • 10^8^ M^−1^s^−1^ is reported

In order to accurately measure the catalytic activity of TtSlyD_FL_ on peptidyl-prolyl *cis*/*trans* isomerization, we carried out Michaelis-Menten studies under equilibrium conditions using NMR lineshape analysis, which is capable of monitoring the rate of exchange between *cis* and *trans* conformations of the peptide substrate. Initially, we benchmarked the method against literature data using the standard suc-ALPF-pNA tetrapeptide (Fig. 7a). We measured a k_cat_/K_M_ value of 1.47 ± 0.05 μM^−1^s^−1^ for the wild-type TtSlyD_FL_, which is in good agreement with previously published data obtained using other methods [20, 21, 23]. For the TtSlyD_ΔIF_ construct, we obtained k_cat_/K_M_ = 0.85 ± 0.01 μM^−1^s^−1^, showing a minor influence of the IF domain. Interestingly, no such effect of the IF domain was detected for *E. coli* SlyD when isomerization of suc-ALPF-pNA was monitored using UV/vis spectroscopy rather than NMR [21].

Having validated the method, we next turned to the longer unmodified peptides of interest here. We opted to use the S2-P25A peptide for these studies because it has the advantage over the S2 peptide that it contains only one proline residue (P29), making data interpretation more straightforward, while it still binds well to both domains (Table 2). The ^13^C shifts of proline in the S2-P25A peptide show the same characteristics as an isolated proline amino acid. By comparing intensities between the *cis* and *trans* forms, the relative population of the *cis* form was found to be 14.5 ± 1 %. We determined apparent rate constants for TtSlyD_FL_ and TtSlyD_ΔIF_ as a function of peptide concentration (Fig. 7b). The resulting k_cat_/K_M_ value was 368 ± 4 μM^–1^s^–1^ for TtSlyD_FL_, which is a factor of 100–1000 higher compared to results obtained previously for various members of the SlyD family using tetrapeptides or unfolded proteins [17, 20, 21, 23, 24], and even exceeds those generally reported for FKBPs and other PPIases [9]. For TtSlyD_ΔIF_ the obtained k_cat_/K_M_ was 248 ± 1 μM^–1^s^–1^, clearly indicating that the IF domain affects the activity of the FKBP domain, similarly to what was observed using tetrapeptide substrates. However, the difference in k_cat_/K_M_ between TtSlyD_FL_ and TtSlyD_ΔIF_ is much smaller than what has been observed using protein substrates [17, 20, 21], and may simply relate to the lower binding affinity of TtSlyD_ΔIF_ relative to TtSlyD_FL_ (in this scenario, K_M_ would be larger for TtSlyD_ΔIF_). In the case of TtSlyD_FL_ we could separate the two parameters, yielding k_cat_ = (0.7 ± 0.1) 10^6^ s^–1^ and K_M_ = 2.0 ± 0.4 mM. Neither value is very precisely determined, but it is obvious from the modest curvature of the Michaelis-Menten plot (Fig. 7b) and the substrate concentrations used here that k_cat_ > > 10,000 s^−1^ and K_M_ > > 100 μM, where the latter value is significantly higher than K_D_. Taken together, these results underline the high catalytic efficiency of TtSlyD when acting on unfolded substrates.

### Mutational probing of catalytically important residues

**Fig. 8:**
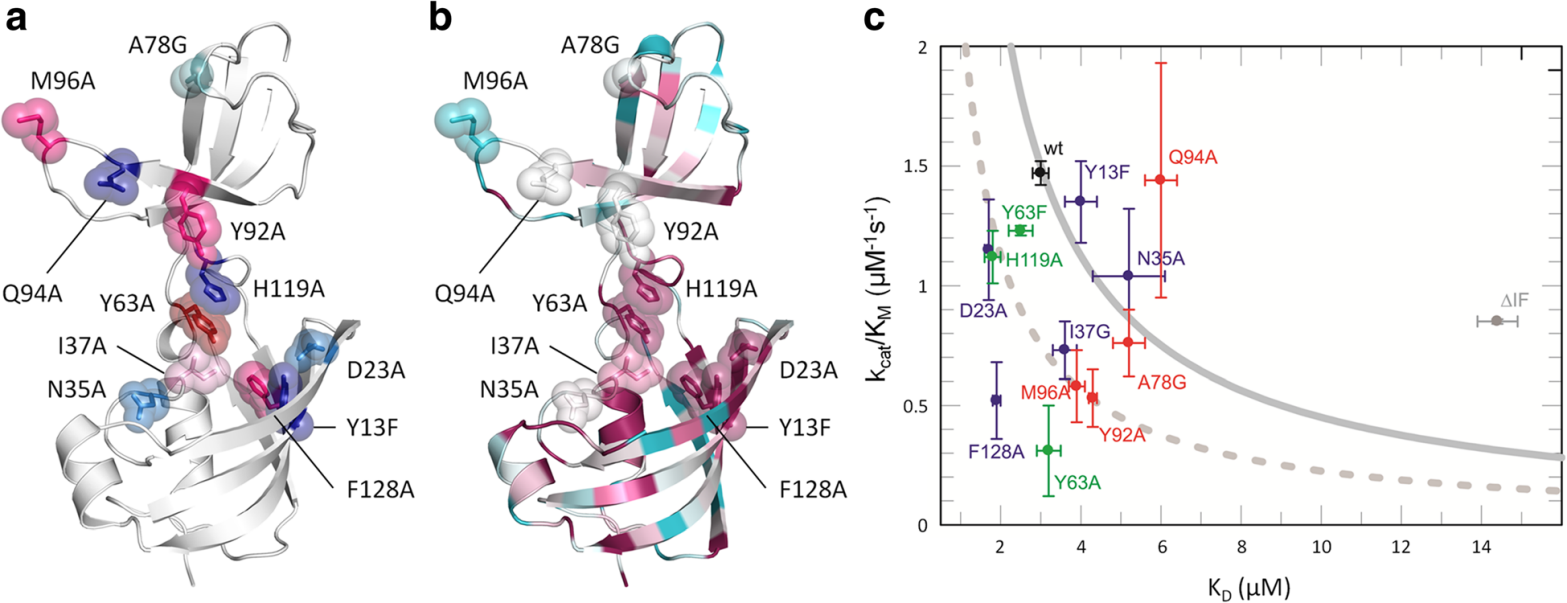
Mutational studies. **a** Mapping of mutated residues on the structure. The side chains of mutated residues are shown in *sticks* and *semi-transparent spheres* for TtSlyD_FL_:S2 molecule A and colored according to activity relative to that of the wild type (see also Table 4): *Dark red*, 0–30 %; *pink*, 31–40 %; *pale pink*, 41–50 %; *pale cyan*, 50–60 %; *bright blue*, 61–80 %; *dark blue*, 81–100 %. Note: Y63 was also mutated to Phe, which caused a reduction to 76 % of that of the wild type. **b** Sequence conservation. Same as in panel A, except that the structure is colored by increasing level of conservation, ramped from *teal* (low conservation) over *cyan*, *white*, and *pink* to *purple* (high conservation). **c** Effect of mutations on activity and binding. k_cat_/K_M_ values from the suc-ALPF-pNA tetrapeptide assay plotted against FK506-binding protein (FKBP) domain-specific K_D_ values of the S2 peptide. Full-length TtSlyD (TtSlyD_FL_) is shown in *black* (labeled wt), TtSlyD constructs with the insert-in-flap (IF) domain replaced by the flap loop from human FKBP12 (TtSlyD_ΔIF_) are shown in *gray* (labeled *ΔIF*), IF domain mutants are shown in *red*, FKBP-domain mutants in *blue*, and linker mutants in *green*. The *solid gray curve* crossing the TtSlyD_FL_ wild-type data point shows the result of varying K_M_ (taken to be equal to K_D_) only. The *dashed gray curve* was generated with k_cat_ = 0.5 · k_cat_ (wild type). The effect of all mutations close to the solid curve (Y13F, N35A, and A78G) can be explained primarily by binding, assuming that the effects on K_D_ and K_M_ are the same. Mutations with data points falling on the dashed curve (Y63F, H119A, D23A, I37G, M96A, and Y92A) can be seen to reduce k_cat_ by a factor of two compared to the wild type, while showing variable binding strength. F128A and Y63A have apparently greater effects on k_cat_, highlighting the importance of these residues

To better understand which residues play an important role for binding and catalysis, we generated a number of mutations in TtSlyD_FL_ and tested their ability to bind the wild-type S2 peptide and catalyze *cis*/*trans* isomerization (Fig. 8 and Table 4). Specifically, we mutated Y63 and H119 of the inter-domain loops; Y13, D23, N35, I37, and F128 of the FKBP domain; and A78, Y92, Q94, and M96 of the IF domain (Fig. 8a, b and Table 4). The affinities for binding of the S2 peptide to these mutants were measured by ITC and the activity determined by NMR using the standard suc-ALPF-pNA tetrapeptide, in order to generate results comparable to previously published activity data. To separate contributions to binding from contributions to catalysis, the NMR-derived k_cat_/K_M_ values were plotted against the FKBP domain-specific K_D_ values of the S2 peptide determined by ITC, and compared to the results expected when a mutation affects only binding or only the turnover rate (Fig. 8c). Although the k_cat_/K_M_ and K_D_ values refer to different substrates, this analysis should provide valuable insights into the relative impact of a given mutation on binding and turnover.

**Table 4:** Results from binding and activity studies on mutated TtSlyD variants

Construct	Mutation	N1	K_D_1 (μM)	ΔH1 (kcal/mol)	–T · ΔS1 (kcal/mol)	N2	K_D_2 (μM)	ΔH2 (kcal/mol)	–T · ΔS2 (kcal/mol)	Activity (kcat/K_M_ 10^6^ M/s)	Relative activity (%)
TtSlyD_FL_	Wild type	0.96 ± 0.03	0.113 ± 0.012	–10.0 ± 0.11	0.7	0.96 ± 0.02	2.93 ± 0.12	–4.9 ± 0.22	–2.5	1.47 ± 0.05	100
	Y13F	0.85 ± 0.03	0.107 ± 0.011	–10.6 ± 0.14	0.5	0.87 ± 0.02	3.94 ± 0.14	–5.9 ± 0.47	–2.6	1.35 ± 0.17	92
	D23A	0.92 ± 0.03	0.075 ± 0.042	–2.0 ± 0.11	1.3	0.88 ± 0.01	1.65 ± 0.15	–6.0 ± 0.11	–2.1	1.15 ± 0.21	78
	N35A	0.98 ± 0.02	0.169 ± 0.032	–9.7 ± 0.11	0.6	1.06 ± 0.05	5.18 ± 0.89	–2.5 ± 0.33	–4.6	1.04 ± 0.28	71
	I37G	0.93 ± 0.05	0.134 ± 0.038	–9.0 ± 0.11	–0.3	0.91 ± 0.03	3.64 ± 0.33	–6.6 ± 0.37	–0.7	0.73 ± 0.12	50
	Y63F	0.81 ± 0.01	0.030 ± 0.003	–11.3 ± 0.11	1.1	0.78 ± 0.02	1.58 ± 0.22	–6.0 ± 0.29	–2.2	1.12 ± 0.11	76
	Y63A	0.80 ± 0.01	0.041 ± 0.007	–9.4 ± 0.06	–0.5	0.84 ± 0.02	3.18 ± 0.29	–4.7 ± 0.15	–2.7	0.31 ± 0.19	21
	A78G	1.08 ± 0.02	0.262 ± 0.023	–8.2 ± 0.06	–0.6	1.09 ± 0.02	5.24 ± 0.43	–2.8 ± 0.19	–4.3	0.76 ± 0.14	52
	Y92A	0.96 ± 0.02	0.254 ± 0.022	–8.7 ± 0.51	–0.1	0.92 ± 0.01	4.33 ± 0.14	–5.3 ± 0.15	–1.9	0.53 ± 0.12	36
	Q94A	1.03 ± 0.03	0.288 ± 0.042	–8.6 ± 0.10	–0.2	0.93 ± 0.02	5.98 ± 0.44	–4.3 ± 0.38	–2.8	1.44 ± 0.49	98
	M96A	1.00 ± 0.01	0.129 ± 0.009	–9.7 ± 0.04	0.4	1.01 ± 0.01	3.86 ± 0.21	–4.4 ± 0.13	–2.9	0.58 ± 0.15	39
	H119A	0.87 ± 0.03	0.060 ± 0.012	–11.1 ± 0.05	0.7	0.82 ± 0.02	2.22 ± 0.28	–6.2 ± 0.47	–2.2	1.23 ± 0.02	84
	F128A	0.88 ± 0.02	0.215 ± 0.022	–9.7 ± 0.36	1.2	0.89 ± 0.02	1.62 ± 0.11	–8.0 ± 0.52	–1.2	0.52 ± 0.16	35
TtSlyD_ΔIF_	Wild type					0.88 ± 0.02	12.23 ± 0.24	–5.9 ± 0.11	0.4	0.85 ± 0.01	58

The affinities and thermodynamic parameters were determined at 20 °C for the wild type S2 peptide using isothermal titration calorimetry, while the activities were determined for the suc-ALPF-pNA tetrapeptide using nuclear magnetic resonance spectroscopy. *TtSlyD*_*ΔIF*_ TtSlyD constructs with the insert-in-flap domain replaced by the flap loop from human FKBP12, *TtSlyD*_*FL*_ full-length TtSlyD

The changes in affinities are about twofold or less in all cases, except for mutations of Y63, where a fivefold increase in the affinity of the IF domain was observed (Table 4), thus supporting the notion of inter-domain cross talk. The mutation with the strongest effect on PPIase activity was Y63A, which retained only 21 % activity (Table 4). It is therefore likely that this residue plays an important role in the catalytic mechanism, in keeping with its ideal position for forming hydrogen bonds with the substrate (Fig. 5c, d). This interpretation is further supported by its high degree of sequence conservation (Fig. 8b) as well as the k_cat_/K_M_ versus K_D_ plot (Fig. 8c). However, the effect on activity is clearly not exclusively due to hydrogen bonds formed by the hydroxyl group, because the Y63F mutant retained as much as 76 % activity (Table 4). Interestingly, the affinity of the FKBP domain was only negligibly decreased for Y63A, but was 1.7-times higher for Y63F with a significantly greater enthalpy and largely unperturbed entropy of binding as compared to the wild type (Table 4). This is similar to what has been observed for binding of FK506 or rapamycin to the equivalent Y82F mutant of FKBP12, where detailed analysis of the binding thermodynamics indicate that the effect of the mutation is due to altered solvation [31].

The second-most detrimental mutations were Y92A, M96A, and F128A, which each retained 30–40 % activity (Table 4). F128 forms part of the hydrophobic binding site (Fig. 5a) and is highly conserved (Fig. 8b), whereas Y92 and M96 are found in the dynamic β8–β9 hairpin of the IF domain and are rather poorly conserved (Fig. 8b). Considering the effect of the binding affinity on k_cat_/K_M_ (Fig. 8c), F128 seems to be as important for catalysis as Y63. Interestingly, while the absence of the IF domain has a positive effect on k_cat_, the Y92A and M96A mutations seem instead to have a negative effect (Fig. 8c). We hypothesize that the effect of these two mutations on the catalytic activity is due to interference with inter-domain cross talk (see “Discussion”).

## Discussion

### Substrate selectivity of the IF domain

ITC experiments revealed that the IF domain binds long peptides with up to nanomolar affinity, and that substantial sequence variation of the substrate can be accommodated through enthalpy-entropy compensation. Notably, we also found that peptides without proline residues can bind to the IF domain, which is in line with a previous study showing that proline-free substrates can inhibit binding of proline-containing substrates to *E. coli* SlyD [24]. Four structures were obtained with 15-residue-long peptides bound to the IF domain (Figs 2 and 4). The peptides bind in the large hydrophobic groove delineated by the 3_10_-helix and β8–β9 hairpin through β-strand augmentation, as was also previously observed for the linker of the uncleaved purification tag in the structure of *E. coli* SlpA [18], and originally predicted based on structural analysis of apo TtSlyD_FL_ [17]. The binding modes are highly variable (Fig. 4), but seem to nonetheless be governed by common recognition principles. It thus appears that the flexible β8–β9 hairpin and the likewise flexible unfolded polypeptide stretch of the substrate are able to structurally adapt to each other, such that one or more (typically two) hydrophobic side chains of the substrate can be sequestered in the quite rigid hydrophobic groove. This highly adaptable binding strategy explains how the IF domain is able to bind extended/flexible polypeptide stretches containing hydrophobic residues in various different sequence contexts. Notably, such stretches are a hallmark of unfolded proteins, and the IF domain therefore seems ideally suited for its function as a folding chaperone.

### Substrate selectivity of the FKBP domain

Most of the peptides tested in the ITC binding studies were found to bind to the FKBP domains of both TtSlyD_FL_ and TtSlyD_ΔIF_, though the affinities were almost invariably highest for the former. Interestingly, as was also found to be the case for the IF domain, the presence of proline residues in the substrate is not a requirement for binding to the FKBP domain in vitro. It is questionable whether such proline-independent binding is physiologically relevant, but this could potentially be the case if the FKBP domain can serve as an auxiliary binding site for the chaperone domain, such as has been suggested for trigger factor [32], or if it can engage non-proline dimerization motifs of cognate interaction partners, such as has been described for human FKBP12 [33]. Five structures were obtained of TtSlyD_FL_ or TtSlyD_ΔIF_ with a 15-residue-long peptide bound to the FKBP domain (Figs 2 and 5). The peptide inserts a *cis*-proline residue into the hydrophobic binding pocket in all cases, except in the TtSlyD_ΔIF_:S3 structure, where a *trans*-valine residue is inserted instead, thus further supporting the conclusion that proline residues are not essential for binding to the FKBP domain. Two peptides, S2 and S2-W23A, adopt β-turn structures with a *cis*-proline in the i + 2 positions. A major role of *cis*-prolines is to enable the formation of type VIa1, VIa2, and VIb β-turns, which all strictly require the presence of a *cis*-proline in the i + 2 position [5, 6]. Based on early computational studies, it was suggested that FKBP12 may be specific for type VIa β-turns [34], whereas the crystal structure of a tetrapeptide–cyclophilin A complex showed the substrates bound as a type VIb β-turn [35]. Taken together, this indicated that there might be a “division of labor” in the cell, with FKBPs and cyclophilins acting on different types of *cis*-proline β-turns. However, our results speak against such a scenario, because both type VIa1 and distorted VIb β-turns are observed in our TtSlyD_FL_:S2, TtSlyD_FL_:S2-W23A, and TtSlyD_ΔIF_:S2-W23A structures, which strongly suggests that FKBPs can catalyze *cis*/*trans* isomerization of prolines present in (at least) both these two types of β-turns.

### A putative mechanism for transition state stabilization at the FKBP domain

**Fig. 9:**
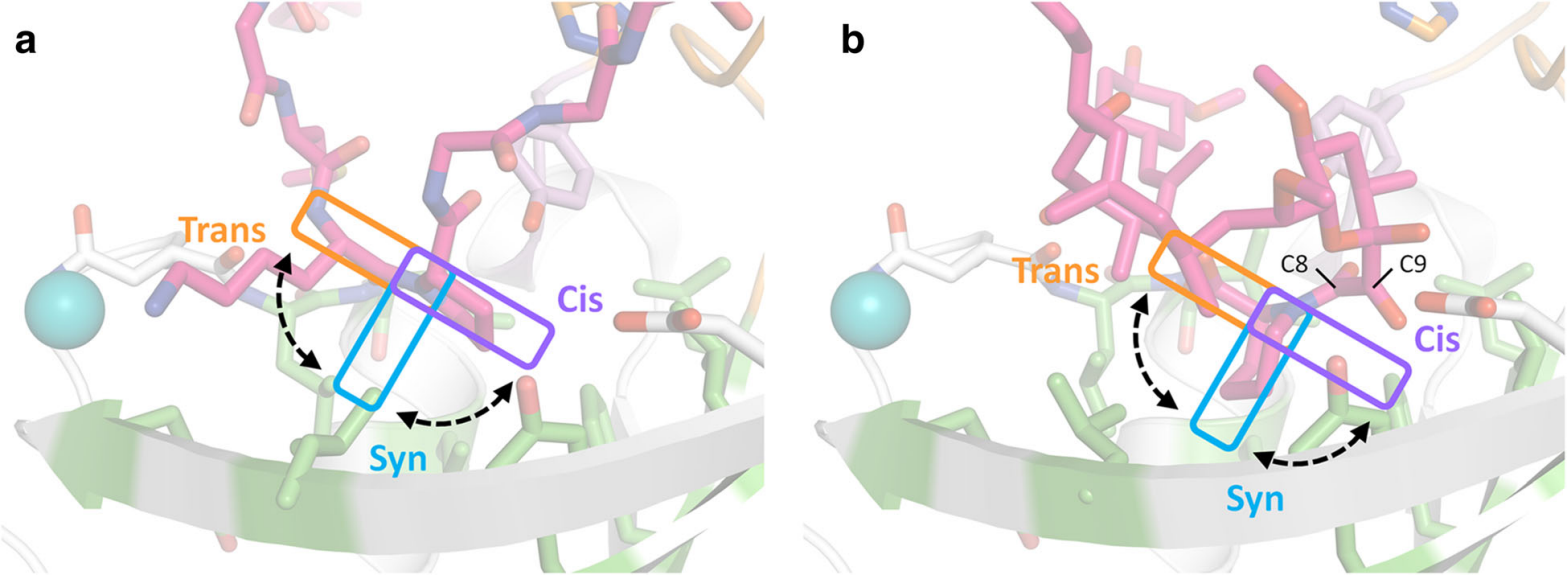
Model for FK506-binding protein (FKBP)-mediated rotation around the peptidyl-prolyl bond. **a** S2-W23A peptide bound to full-length TtSlyD (TtSlyD_FL_) molecule D (β-turn type VIb-like binding mode). The color scheme is the same as for Fig. 5, except that the colors are more subdued. The hypothesized rotations of the P29_S2_ proline residue are indicated. We suggest that the residue found immediately N-terminal to the peptidyl-prolyl bond are kept anchored during catalysis through β-strand type hydrogen bonds and side chain interactions with the hydrophobic pocket (see also Fig. 5c, d and Fig. 6a). The C-terminal part, including the side chain of the proline, then rotates from the *trans* to the *cis* form via the twisted transition state (*syn* form) or vice versa, as indicated. Notably, the model predicts that the proline side chain penetrates deepest into the pocket in the twisted transition state, which may confer preferential stabilization of this form over the ground states. **b** Binding of FK506 to TtSlyD_FL_. The orientation and color scheme are the same as in panel a. The pipecolinyl ring is in the same position as expected for the proline side chain when in the *syn* form, which supports the notion that FK506 can be viewed as a mimic of the twisted transition state

The catalytic mechanism of PPIases does not involve any bond formation or breakage, but hinges instead on rotation around the peptidyl-prolyl bond, which is at least partially mediated by preferential stabilization of the twisted transition state [9, 10]. In the case of the cyclophilins, there has been some debate as to whether it is the part N- or C-terminal to the peptidyl-prolyl bond that rotates [36–38]. The FKBP field has not seen a similar debate, which is probably mainly due to a dearth of informative substrate complexes. However, on the basis of the structures presented here, we find it most likely that FKBPs operate with C-terminal rotation (Fig. 9a), though we concede that a definitive conclusion regarding this question will require that structures representing the *trans* form are also obtained. The basis for our assertion is that the two residues N-terminal to the peptidyl-prolyl bond are anchored in the same way in both the type VIa1 and VIb-like binding modes through two β-strand type hydrogen bonds supplemented by side chain interactions with the hydrophobic binding pocket (Fig. 5c, d and Fig. 6a), whereas the residues found C-terminal to the peptidyl-prolyl bond adopt very different positions in the VIa1 and VIb-like β-turns, suggesting that this part would have more freedom to rotate during catalysis (Fig. 5c, d). In line with this, it has been shown that FKBP12 exhibits higher sequence specificity towards the residue that immediately precedes the proline than the one that immediately follows it [39]. In addition, we also found that FK506 forms two hydrogen bonds that mimic the β-strand type hydrogen bonds formed by the peptides (Fig. 6a). Moreover, this is not unique to TtSlyD:FK506, but is also commonly observed in structures of FKBP12:inhibitor complexes [40]. Indeed, based on such structures, it was already predicted that peptides would bind via two β-strand type hydrogen bonds in the same manner, as we have now observed for TtSlyD [40]. Furthermore, the large appendage at the C26 position of FK506 overlaps with the side chain of M27_S2_, that is, the residue that precedes the proline by two positions. We therefore conclude that binding of FK506 involves several interactions that mimic those of the N-terminal part of a bound polypeptide, which supports the notion that this is the part that is kept anchored during catalysis.

Interestingly, a consequence of C-terminal rotation is that the proline side chain would reach considerably deeper into the binding pocket and interact more extensively with it in the *syn* form (ω ~90°) relative to the *cis* and *trans* forms (Fig. 9a), which could be a key factor in conferring preferential stabilization of the twisted transition state. In keeping with this notion, it is well established that the hydrophobic environment of the binding pocket is critically important for the catalytic mechanism [41–45], as is also supported by our finding that the F128A mutation in the bottom of the binding pocket reduced the catalytic activity to 35 % of that of the wild type. If, on the other hand, TtSlyD mediates N-terminal rotation, then the proline side chain would remain throughout catalysis in the shallow position observed in the present structures.

### FK506 as a potential mimic of the twisted transition state

FK506 has been proposed to mimic the twisted transition state with the pipecolinyl ring being a surrogate of the proline side chain, and the roughly orthogonal α-keto carbonyl group at C9 being a surrogate of its twisted backbone carbonyl group [27, 28]. The situation is, however, complicated by the fact that a *trans* amide carbonyl is found at the C8 position between the ring and the α-keto carbonyl, which could also serve as a mimic of the proline carbonyl group. Interestingly, we found that Y63 hydrogen bonds to both the C8 carbonyl oxygen atom of FK506 and the *cis*-proline carbonyl atoms of peptides bound as a type VIb-like β-turn (Fig. 6b). It may therefore be argued that FK506 mimics aspects of the type VIb-like *cis*-state, though it should be pointed out that the angles of the Y63-carbonyl hydrogen bonds are markedly different in the two cases, as a consequence of the different positions of the two carbonyl groups (Fig. 6b). A probably more significant observation is that the pipecolinyl ring is roughly orthogonal to the side chains of the *cis*-prolines of the bound peptides regardless of their adopted binding mode (Fig. 6b), and penetrates considerably deeper into the binding pocket. Indeed, under the premise that catalysis proceeds through C-terminal rotation around the peptidyl-prolyl bond, this finding strongly supports the notion that FK506 mimics the twisted transition state of the substrate (Fig. 9b).

### The role of tyrosine-63 in the catalytic mechanism

In our mutational analysis of the binding site, the Y63A mutation was found to be the most severe, with a residual activity of only 21 %. The corresponding Y82 residue in FKBP12 has been proposed to aid in catalysis by forming a hydrogen bond to the proline imide nitrogen, thereby lowering the rotational barrier of the peptidyl-prolyl bond [46]. In line with this, we found that the hydroxyl group of Y63 is indeed within hydrogen-bonding distance of the imide nitrogen of cis-P29_S2_ in both the type VIa1 and VIb-like peptide binding modes, if applying a generous cut-off (the distances are 3.2–3.4 Å for the former binding mode and 3.5–3.6 Å for the latter). The hydroxyl group of Y63 is furthermore within hydrogen-bonding distance of the backbone nitrogen of F30_S2_ and the carbonyls of M27_S2_ and I31_S2_ in the case of the type VIa1 binding mode (Fig. 5c), and of the P29_S2_ carbonyl group in the case of the type VIb-like binding mode (Fig. 5d), which might implicate it in appropriately orienting the substrate in the binding site. The functional role of Y63 is, however, not entirely dependent on the hydroxyl group, given that the Y63F mutant retained as much as 76 % activity. In line with this, a mutational study on human FKBP12 where the equivalent Y82 residue was replaced by each of the other 19 proteinogenic amino acids showed that aromatic residues, arginine, and proline retained or even improved activity, whereas most other residues caused a substantial reduction [43]. Furthermore, similar results have also been obtained for the equivalent Y100 residue in *Plasmodium vivax* FKBP35, except that arginine and, in particular, proline replacements were less well tolerated [22]. Finally, it may be noted that although Y63 is highly conserved, a phenylalanine is found in its place in some catalytically active FKBPs, for example, *E. coli* SlpA [47]. It is therefore clear that other interactions must be able to partially substitute for any functionally important hydrogen bonds formed by the hydroxyl group, for example, CH–π, CH–O, or CH–N hydrogen bonds and/or van der Waal interactions. In relation to this point, it may be noted that several putative CH–O hydrogen bonds between FKBPs and bound inhibitors have been identified in FKBP:inhibitor structures [25, 48], and that an NMR analysis of aromatic ring flips has identified a hydrogen bond between the ζ hydrogen of F46 and rapamycin in the FKBP12:rapamycin complex [49].

Comparisons of the crystal structures obtained for TtSlyD_FL_ indicate that Y63 is found in a highly mobile hinge region, which we further confirmed by aromatic ^1^H–^13^C NMR studies on apo TtSlyD. Furthermore, the flap loop of FKBP12, which encompasses the equivalent Y82 residue, has also been shown to be flexible or mobile [50–53]. This mobility of Y63 can be expected to enable a certain level of dynamic remodeling of the binding site, which could be important for allowing it to optimally interact with structurally different substrates and/or for adapting to their motions during catalysis. In conclusion, the role of Y63 is not yet fully clarified, but likely hinges on a combination of its ideal position for interacting with the substrate and its high level of mobility.

### Evidence for inter-domain cross talk

It has been reported that there is cross talk between the IF and FKBP domains in the sense that binding to one domain affects dynamics [29], substrate affinity [23, 30], and stability [54] of the other. Here we show that 15-residue-long peptides can be bound to each of the two domains at the same time, and that deletion of the IF domain results in reduced affinity and activity of the FKBP domain of TtSlyD. Interestingly, the reduction in affinity stems mainly from less favorable entropy, suggesting that it may be a consequence of the enhanced dynamics of the FKBP domain that reportedly results from substrate binding at the IF domain [29]. We furthermore show that mutating Y63 or H119, which are part of both the inter-domain connectors and the active site of the FKBP domain, increases the affinity of the IF domain up to fivefold, and that Y63 adopts different orientations depending on the substrate, which interestingly correlates with different positions of the IF and FKBP domains relative to each other (Fig. 6c). This may suggest that Y63 and the connector loops may be important for inter-domain cross talk, but more studies will be needed to confirm this. Finally, we show that mutating Y92 or M96 in the IF domain to alanine reduces the PPIase activity to 36–39 % relative to the wild type without substantially affecting affinities, indicating that these mutations may affect the dynamics of catalytically important FKBP residues. In conclusion, our results clearly support the notion of inter-domain cross talk in SlyD, and highlight the need for further experiments to pinpoint the molecular mechanisms underlying this phenomenon.

### Catalytic activity

Our NMR lineshape analysis of TtSlyD_FL_ activity on the commonly used tetrapeptide (suc-ALPF-pNA) yielded k_cat_/K_M_ = 1.5 10^6^ M^−1^s^−1^, which is in line with previously published values obtained for *E. coli* SlyD using a fluorescence-based assay (0.25–1.10 10^6^ M^−1^s^−1^ [20, 21]). The presence of the IF domain was found to have a small effect on the activity towards tetrapeptides, which contrasts with previous studies, where no effect was observed [21]. This effect probably relates to the effect of the IF domain on the affinity of the FKBP domain. For partially folded proteins, similar k_cat_/K_M_ values (0.6–1.2 10^6^ M^−1^s^−1^) but much higher affinities have been reported [19, 23]. However, in these cases the absence of the IF domain decreased the activity by a factor of 100–200 [17, 20, 21]. Our studies using 15-residue-long peptides paint a different picture. These peptides bind with affinities that are similar to those of partially folded proteins, and thus much higher than those of tetrapeptides. However, the NMR-derived activity for the S2-P25A peptide is 3.7 10^8^ M^−1^s^−1^, and thus a factor of 100-times higher than for both tetrapeptides and partially folded proteins. Indeed, the activity is of the same order as that of super-efficient enzymes, with k_cat_/K_M_ in the range 10^8^–10^10^ M^−1^s^−1^, whose activity is considered diffusion-limited [55]. This might appear surprising given that TtSlyD does not have a defined optimal substrate, but rather catalyzes the isomerization of a broad range of proline-containing peptides that form variable contacts with TtSlyD. However, it can be explained on the grounds that the catalytic efficiency of TtSlyD is not hindered by substrate specificity. The IF domain was found to have a definite but small effect on the activity (2.5 10^8^ M^−1^s^−1^ in the absence of the IF domain), similar to what we observed for suc-ALPF-pNA. Our Michaelis-Menten analysis of TtSlyD_FL_ with the S2-P25A peptide revealed k_cat_ = 700,000 s^–1^ and K_M_ = 2000 μM, a result that highlights the high efficiency of TtSlyD. For comparison, the homologous human FKBP12 yields a k_cat_ above 10,000 s^−1^ with suc-ALPF-pNA as a substrate (U Weininger, unpublished data), again indicating high catalytic efficiency. This contrasts with k_cat_ values determined for partially folded protein substrates, which have been estimated to be around 1 s^–1^ [24].

Our findings can be summarized as follows: a high catalytic rate constant of k_cat_ > 10,000 s^−1^ is obtained with both S2-P25A and the suc-ALPF-pNA tetrapeptide as substrates. However, because the longer S2-P25A peptide can make more contacts with the protein (enthalpy), or retain more flexibility (entropy) in the bound state, it has lower K_D_ and K_M_ values, resulting in higher activity (k_cat_/K_M_). By contrast, with partially folded proteins as substrates, one observes a very low k_cat_ (which might be coupled to folding), a strong impact of the IF domain, and a lower overall activity than what is found using peptide substrates. Taken together, the available data suggest that while SlyD can isomerize various kinds of substrates, including partially folded or unfolded proteins, it is most efficient when acting on unfolded polypeptide chains.

## Conclusions

We have shown that 15-residue-long unmodified peptides bind to TtSlyD with affinities that are similar to those of partially folded proteins, but considerably higher than estimated for the chemically modified tetrapeptides that are typically used for functional studies on FKBPs. We therefore conclude that long unmodified peptides are better mimics of unfolded protein substrates than the classical tetrapeptides. We have furthermore shown that the enzymatic activity towards the 15-residue-long S2-P25A peptide is much higher than for both tetrapeptides and partially folded proteins, implying that TtSlyD is most efficient when acting on unfolded proteins. In addition, we have presented several structures of TtSlyD in complex with 15-residue-long peptides, which represent the first structures of an FKBP protein in complex with long unmodified peptides. These structures show that unfolded polypeptides bind to the IF domain in a highly adaptable fashion involving β-strand augmentation and hydrophobic interactions, which agrees well with the low sequence specificity of this domain. Furthermore, they also show that substrates can bind to the FKBP domain as both types VIa1 and VIb-like β-turns, indicating that FKBPs are functionally more versatile than previously appreciated. Based on a comparison of our structures, we have proposed a novel general model for the catalytic mechanism of FKBPs that involves C-terminal rotation around the peptidyl-prolyl bond mediated by stabilization of the partially rotated transition state in the hydrophobic binding site. Our results furthermore establish that Y63 (Y82 in human FKBP12) is important for the catalytic activity of TtSlyD. Why this is the case is still not entirely clear, but it likely relates to its ideal position for interacting with substrates bound to the FKBP domain and its high mobility. Finally, we have found further evidence for the previously reported phenomenon of inter-domain cross talk between the IF and FKBP domains, and propose that Y63 may also be important in this context. To sum up, we show in great detail how the IF and FKBP domains recognize unfolded protein mimics, and provide novel insights into the general catalytic mechanism of FKBPs.

## Methods

### Reagents

Isopropyl-β-D-thiogalactopyranoside (IPTG) was purchased from Anatrace (Maumee, OH, USA). Lysogeny broth medium was from Becton Dickinson (Franklin Lakes, NJ, USA) and terrific broth was from Formedium (Norfolk, UK). The peptides used in this study had amidated C-termini and were, with two exceptions, purchased from GL Biochem Ltd (Shanghai, China). The exceptions were the S2-P25A peptide with a selectively labeled (N^15^C^13^) proline residue, which was from JPT (Berlin, Germany), and suc-ALPF-pNA, which was obtained from Bachem (Bubendorf, Switzerland). The sequences of all used peptides are given in Table 1. α-Lactalbumin was from Sigma-Aldrich (St. Louis, MO, USA) and the permanently unfolded state of RCM-α-lactalbumin was prepared by reduction and carboxymethylation, as described [56]. Crystallization reagents were from Qiagen (Germantown, MD, USA). All other chemicals were of analytical grade and obtained from Sigma-Aldrich, unless otherwise stated.

### Protein expression and purification

Full-length SlyD from *T. thermophilus* (UniProt Q5SLE7), TtSlyD_FL_, and the chimeric construct TtSlyD_ΔIF_, where the IF chaperone domain and inter-domain linkers (residues 65–125) are replaced by the flap loop from human FKBP12 (UniProt P62942 residues 84–96), were expressed and purified as previously described [17], except that 20 mM 4-(2-hydroxyethyl)-1-piperazineethanesulfonic acid (HEPES), 100 mM NaCl, pH 7.5, was used as final buffer. All recombinant TtSlyD constructs contained a C-terminal hexahistidine tag (His153 to His158 in TtSlyD_FL_ and His105 to His110 in TtSlyD_ΔIF_) with an additional small linker before the tag (Pro150-Ser151-Gly152 in TtSlyD_FL_ and Pro102-Ser103-Gly104 in TtSlyD_ΔIF_) to facilitate purification and on-column refolding using immobilized metal affinity chromatography. We also made an additional construct with a thrombin cleavage site between the SlyD gene and the C-terminal hexahistidine tag for an ITC control experiment. Here, only four additional C-terminal residues (Leu150-Val151-Pro152-Lys153) remain attached to the purified protein construct after tag cleavage. The correct identity of the expressed and purified proteins was verified by electrospray mass spectrometry. ^15^N isotope-labeled NMR samples were produced using M9 minimal media based on ^15^NH_4_Cl as nitrogen source (Spectra Stable Isotopes, USA) and supplemented with vitamin mixture.

### Isothermal titration calorimetry

ITC measurements were performed on iTC200 or VP-ITC instruments (GE Healthcare, Chalfont St. Giles, UK). The calorimetric cell (with a total cell volume of 220 μl in the iTC200, or 1400 μl in the VP-ITC) contained 50–200 μM TtSlyD_FL_ dissolved in 20 mM HEPES, 100 mM NaCl, pH 7.5. Peptide samples of 700–2000 μM were titrated into the cell at 20 °C (VP-ITC) or 25 °C (iTC200). The heat generated after each ligand injection was obtained by integration of the calorimetric signal. Resulting binding isotherms were analyzed according to a one-site or two-site binding site model using the Origin software (OriginLab Corp., Northampton, MA, USA).

### NMR spectroscopy

NMR samples contained TtSlyD in 20 mM HEPES, 100 mM NaCl, pH 7.5, and 10 % (v/v) D_2_O. All experiments were performed at a static magnetic field strength of 14.1 T and a temperature of 25 °C. The spectra were processed with NMRpipe [57] and analyzed with NMRview [58], except one-dimensional spectra, which were processed and analyzed using VNMRJ (Agilent, Inc.). Lineshape analysis was performed using MATLAB.

### Binding studies using NMR spectroscopy

In order to study binding of the S2-P25A peptide to TtSlyD_FL_, 100 μM of ^15^N-labeled TtSlyD_FL_ was titrated with unlabeled peptide up to a total peptide concentration of 280 μM (at this point the protein was diluted to a concentration of 72 μM). Amide signals from the free and bound state were found to be in fast or intermediate exchange (depending on the chemical shift difference). Signals in fast exchange were tracked using a weighted mean ^1^H and ^15^N chemical shift difference between the free and bound states. Residues with a chemical shift difference >0.1 ppm and residues completely broadened because of intermediate exchange were used to map the effect of binding on the structure. In order to assign the two binding events detected by ITC to the individual domains of TtSlyD_FL_, residue-specific chemical shift titration curves were obtained for residues in fast exchange in both the ^1^H and ^15^N dimensions, and compared to simulated titration curves generated using the ITC-derived K_D_ values. One should bear in mind that since the substrate can exist in two forms, *cis* and *trans*, the determined K_D_ value is an effective average over the two K_D_ values, 1/K_D_ = (1/K_D,cis_ + K/K_D,trans_)/(K + 1), where K is the equilibrium constant K = [*trans*]/[*cis*].

### Activity studies using NMR spectroscopy

For tetrapeptide activity studies, one-dimensional ^1^H spectra of 500 μM suc-ALPF-pNA tetrapeptide containing 2 μM, 4 μM, or 6 μM of TtSlyD were recorded for the different mutants [59]. Apparent exchange rates between the *cis* and the *trans* states were derived by lineshape analysis of one methyl resonance of the leucine residue, which displays different chemical shifts for the *cis* and *trans* states. k_cat_/K_M_ values were subsequently determined by linear regression of the exchange rate versus enzyme concentration. Because the k_cat_/K_M_ values are determined by lineshape analysis in an equilibrium experiment, they are not expected to be directly comparable to the k_cat_/K_M_ values obtained from traditional initial-rate stopped-flow experiments. Nevertheless, our validation experiments (see below) give the same results within the range of errors. Furthermore, the k_cat_/K_M_ values obtained for a series of mutant enzymes can be directly compared as a measure of relative efficiency, even if the values are not directly comparable to the ones obtained in a traditional experiment.

Activity studies using the 15-residue-long S2-P25A peptide utilized a sample with a uniformly ^13^C-labeled proline residue. ^1^H–^13^C HSQC experiments were used to separate the peptide signals in two dimensions, such that the *cis* and *trans* forms could both be detected without overlap. Further analysis focused on the signals from the β and γ position, because they displayed the largest chemical shift differences and appear in isolated regions of the ^1^H–^13^C HSQC spectrum. One-dimensional ^1^H slices of the β and γ protons for the *cis* and *trans* forms were extracted from the two-dimensional spectrum, and lineshape analysis was applied. Michaelis-Menten-like studies were performed using a fixed concentration of TtSlyD_FL_ of 0.2 μM and peptide concentrations ranging from 10 to 1000 μM.

### Crystallization of TtSlyD in complex with ligands

TtSlyD_FL_ and TtSlyD_ΔIF_ were concentrated to 30–60 mg/ml. For complex formation, the protein was typically incubated with a threefold excess of peptide or the compound FK506 for at least 1 hour prior to crystallization. Crystals were grown by the sitting-drop vapor diffusion method at 4 °C in 96-well crystallization plates using total drop volumes of 0.2–0.3 μl. Crystallization conditions varied depending on which ligand was used, but in all cases the pH was in the range of 5.0–8.0 and polyethylene glycol (PEG) was used as precipitant (Table 5). The crystals were then cryoprotected by the addition of 20 % glycerol to the original crystallization condition, and flash frozen in liquid nitrogen, after which X-ray diffraction data were collected at the Diamond or SOLEIL synchrotrons.

**Table 5:** Crystallization conditions

Complex	Precipitant	Buffer	Salt
TtSlyD_FL_:T1	13.2 % PEG1500	0.1 M HEPES pH 7.5	0.05 M NaCl
TtSlyD_FL_:S2	20 % PEG3350	0.1 M Bis Tris pH 5.5	0.2 M (NH_4_)_2_SO_4_
TtSlyD_FL_:S2-W23A	19 % PEG3350	8 % Tacsimate pH 5.0	–
TtSlyD_FL_:S2-plus2	20 % PEG6000	0.1 M Citric acid pH 5.0	–
TtSlyD_FL_:FK506	25 % PEG 3350	0.1 M Bis Tris pH 5.5	0.2 M MgCl_2_
TtSlyD_∆IF_:S2-W23A	28 % PEG400	0.1 M HEPES pH 7.5	0.2 M CaCl_2_
TtSlyD_∆IF_:S3	25 % PEG6000	0.1 M Tris HCl pH 8.0	0.2 M CaCl_2_
TtSlyD_∆IF_:FK506	20 % PEG6000	0.1 M CH_3_O_2_Na pH 5.0	0.2 M ZnCl_2_

No additives were used except for TtSlyD:T1, where 11 % glycerol was present in the crystallant

### X-ray structure determination

We used XDS and XSCALE [60] for processing our X-ray diffraction data sets (Table 3). With the exception of TtSlyD_∆IF_:S2-W23A and TtSlyD_∆IF_:S3, all structures represent different crystal forms (Table 3), which is not unexpected considering the wide variability in ligands and crystallization conditions. For TtSlyD_FL_, phasing was achieved using a previously determined structure of the protein [PDB: 3LUO] as the search model for molecular replacement in the Phaser program [61] of the Phenix suite [62]. Generally, a good solution required that the FKBP and IF domains were placed separately. For TtSlyD_∆IF_:S2-W23A, we noticed a quite strong anomalous signal in the data, and we therefore carried out SAD phasing in Phenix AutoSol [63] instead of molecular replacement. The phasing statistics were as follows: Bayes-CC = 57.19 ± 7.07 and FOM = 0.492. The structure revealed that the signal stems from a number of bound Ca^2+^ ions (the crystal was grown in the presence of CaCl_2_), as well as a metal ion coordinated by six histidines, which we have interpreted as a co-purified Ni^2+^ ion (Additional file 3). Phases for the other two TtSlyD_ΔIF_ structures were obtained by molecular replacement in Phaser (though a considerable anomalous signal was also present in these data sets). For all structures, refinement was carried out using iterative cycles of manual rebuilding in Coot [64] and maximum likelihood refinement in Phenix refine version 1.8 [62]. Refinement statistics are shown in Table 3 along with validation results (Ramachandran plot and clash score) from Molprobity version 4.3 [65]. We generally used isotropic B-factor refinement with translation libration screw (TLS) restraints, with one TLS group for each protein and peptide chain (and no groups for FK506 or solvent). The exceptions to this are the 1.4 Å TtSlyD_FL_:T1 structure where we used anisotropic B-factor refinement without TLS, and the 1.75 Å TtSlyD_∆IF_:S2-W23A structure, where we used a combination of isotropic B-factor refinement with TLS (protein and peptide chains) and anisotropic B-factor refinement without TLS (ions).

### Protein structure analysis

Analysis of hinge regions was performed using DynDom version 2.0 [66]; protein:substrate interfaces were analyzed using the PISA program version 1.51 [67]; and conservation analysis was done using ConSurf [68]: specifically, an alignment of 150 proteins (25–95 % identical to TtSlyD) from UniRef-90 was used for coloring the structure by sequence conservation. All protein structure figures were made using the PyMol program [69].

## Additional files

Additional file 1:
^1^H–^13^C transverse relaxation optimized spectroscopy hetero single quantum coherence (TROSY-HSQC) spectra of 1 mM apo TtSlyD at 25 °C. **a** The Fδ,ε/Yδ region. **b** The Yε region. δ positions are ^13^C-labeled by 1-^13^C_1_-glucose and shown in black. ε are ^13^C-labeled by 2-^13^C_1_-glucose and shown in red. All expected resonances have been identified and assigned, with the exception of Y63, which presumably is line-broadened beyond detection due to conformational exchange. (PNG 363 kb) Additional file 2: Chloride binding site. Two examples are shown: TtSlyD_FL_:S2-W23A chain A (left) and TtSlyD_FL_:FK506 chain B (right). TtSlyD is white, the peptide is pink, chloride is turquoise, and water molecules are red. The transparent spheres indicate the van der Waal radii of the chloride ions as given by PyMol. Distances up to 3.4 Å (first coordination sphere of chloride [70]) are indicated with black dashes and longer distances are gray. The annotation as chloride was based on the binding mode and the electron density maps (including the anomalous difference Fourier map where weak density for the ion could be recognized). The backbone nitrogen atoms of L30 and A148 interact with the ion. The distance here is ~3.2 Å for all structures, which is the most commonly found coordination distance for chloride [70]. The side chain of N35 also contacts the ion, though the distances here are longer and more variable. When an S2, S2-W23A, or S3 peptide is bound, the Nζ atom of K28_S2_/K16_S3_ is within 3.1–3.6 Å of the ion (left panel). The number of ligands is typically three to five, which is also the most common range for chloride in general [70]. Part of the coordination sphere is made up of water molecules that vary somewhat in exact position. Note that the chloride ion is present under both low and moderately high salt concentrations (Table 5), indicating that it is unlikely to be a crystallization artifact. (PNG 975 kb) Additional file 3: Metal coordination. Binding of a nickel ion in TtSlyD_ΔIF_:S2-W23A **a** and a zinc ion in TtSlyD_ΔIF_:FK506 chain A **b**. Native protein residues are white and the His-tag is pale pink, except for symmetry-related mates where the protein is dark gray and the His-tag is purple. Chloride is turquoise, the putative nickel ion is green, and the zinc ion is slate. Two views are shown: an overview in the same orientation as in Fig. 2 (left panels) and a zoomed view, which is reoriented to better show the coordination geometry (right panels). In the latter view, coordination bonds are indicated with solid black lines and residues are labeled (the corresponding residues of the TtSlyD_FL_ construct are indicated in parentheses). Asterisks denote residues in symmetry-related mates. The putative nickel ion is octahedrally coordinated by six histidine residues: three native residues, two from the His-tag, and one from the His-tag of a symmetry-related mate. The same coordination pattern is seen in TtSlyD_ΔIF_:S3 and in a previously determined structure of TtSlyD_FL_ [PDB: 3CGM] [17]. The zinc ion is tetrahedrallly coordinated by the same three native histidines residues that are also used for coordinating the putative nickel ion, as well as a histidine from a symmetry-related mate. A similar pattern is seen in another of the previously determined TtSlyD_FL_ structures [PDB: 3LUO] [17], though here the symmetry-related histidine residue is replaced by a water molecule, which is bound at a somewhat different angle. (PNG 2098 kb) Additional file 4: Electron density maps for the S2 peptide bound to the IF domain. **a** Electron density map for the S2 peptide bound to the IF domain of molecule A of the 2.9-Å TtSlyD_FL_:S2 structure. The IF domain is blue, the S2 peptide is pink, and the 2Fo-Fc electron density map for the peptide is gray (contoured at 1 σ). In spite of the modest resolution, the peptide is well defined in the electron density map, and could therefore be confidently modeled. **b** The S2 peptide bound to molecule B in the TtSlyD_FL_:S2 structure. This peptide is not very well defined in the electron density map, and could therefore not be modeled with high confidence. However, it is nonetheless clear that it is bound in a very different manner than the peptide bound to molecule A (see also Fig. 4b, c). (PNG 2247 kb) Additional file 5: Structural changes in the IF domain upon substrate binding. **a** Overlay of the IF domain of the three TtSlyD_FL_ molecules of the TtSlyD_FL_:FK506 structure, which is the only structure we obtained where no substrate was bound to the IF domain. Molecule A is pale green, molecule B is wheat, and molecule C is pale slate. The side chains of residues lining the hydrophobic groove are shown in sticks. Note that there is very little variation in the positions and conformations of these residues, except that some variability can be observed for V74, L103, and F117. **b**–**d** Overlay of the substrate-free IF domain of TtSlyD_FL_:FK506 molecule B with three substrate-bound structures: TtSlyD_FL_:S2 molecule A (panel B), TtSlyD_FL_:S2-W23A molecule C (panel C), and S2-plus2 (panel D). TtSlyD_FL_:FK506 molecule B is colored wheat, as in panel C, and the substrate-bound structures are colored as in Fig. 4. Binding does not notably affect the hydrophobic binding groove. Thus, only V74, L103, and F117 display clear variability between the substrate-bound structures and the TtSlyD_FL_:FK506 molecule B apo structure, and not more pronouncedly so than between the three individual TtSlyD_FL_ molecules in the TtSlyD_FL_:FK506 apo structure (panel A). (PNG 2763 kb) Additional file 6: Mapping of the interaction faces between the IF domain and the bound peptides for representative structures. **a** The S2 peptide bound to the IF domain of molecule A in the TtSlyD_FL_:S2 structure. The peptide is shown in sticks and TtSlyD_FL_ in semi-transparent surface representation. Residues forming van der Waal interactions, hydrogen bonds, or salt bridges between TtSlyD and the peptide are colored as in Fig. 4, while all non-interacting residues are dark gray in both protein and peptide. Selected residues in the peptide are labeled. **b** The S2-W23A peptide bound to the IF domain of molecule C in the TtSlyD_FL_:S2-W23A structure. **c** The S2-plus2 peptide bound to the IF domain in the TtSlyD_FL_:S2-plus2 structure. **d** The T1 peptide bound to the IF domain in the TtSlyD_FL_:T1 structure. **e** Interaction lists for the structures shown in panels a–d. Hydrogen bonds (≤3.5 Å) and salt bridges (≤4.0 Å) detected using the PISA program are listed along with van der Waal interactions between carbon and carbon/sulfur atoms (≤4.0 Å). The specific atoms mediating the interactions between residues are specified for the hydrogen bonds and salt bridges. Interacting residues are colored as in panel a–d. Asterisks designate β-strand hydrogen bonds augmenting the β8–β9 hairpin, and dagger symbols designate residues that are poorly defined in the electron density map (real-space correlation coefficient < 0.7). Interactions with symmetry-related mates are omitted. There are few hydrogen bonds and salt bridges apart from the β-strand interactions, while there are many peripheral van der Waal interactions that do not involve the hydrophobic groove, most of which involve the β8–β9 hairpin instead. (PNG 1947 kb) Additional file 7: Structural changes in the FKBP domain upon substrate binding. **a** Superimposition of molecule A from the TtSlyD_FL_:S2-W23A structure, which represents the type VIa1 binding mode, on the FKBP domain of TtSlyD_FL_:S2-plus2, which represents the substrate-free form. The substrate-bound structure is colored as in Fig. 5 and the apo structure is colored slate. **b** Superimposition of molecule D from the TtSlyD_FL_:S2-W23A structure, which represents the type VIb-like binding mode, on the apo form (same color scheme as in panel a). **c** Superimposition of molecule B from the TtSlyD_FL_:FK506 structure, which represents the FK506 binding mode on the apo form (same color scheme as in panel a). Overall, there is substantial variation in the positions of Y63, and a more modest variation in the position of the loop encompassing L36 and I37 between the substrate-bound forms and the apo form. There is no overall positional shift in any of the other residues in the hydrophobic binding pocket, though some variability in the side chain configurations of D23, L27, and L126 can be detected, which may or may not be linked to substrate binding. (PNG 1394 kb) Additional file 8: Non-canonical binding to the FKBP domain. **a** Binding of the T1 peptide to the FKBP domain in the 1.6-Å structure of TtSlyD_FL_:T1. The model is colored as in Fig. 5, and the 1 σ 2Fo-Fc electron density map is shown in gray around the peptide and key binding site residues. Two views are shown – an overview displaying putative hydrogen bonds (black dashes) on the left and a focused view of P65 of the peptide (P65_T1_) on the right. Although the electron density map is generally of high quality for this structure (as exemplified by Y63 in the left panel), it is quite poor for the peptide bound to the FKBP domain, suggesting that the occupancy is low and/or the flexibility is high. This agrees well with the binding studies, which showed that the T1 peptide binds with low affinity. Nonetheless, P65_T1_ could be placed fairly confidently in the center of the binding site where it is bound in the *cis* form. Notably, it is bound in the opposite direction as compared to the S2 and S2-W23A peptides. **b** Binding of the S3 peptide to the FKBP domain in the 2.0-Å structure of TtSlyD_ΔIF_:S3. Depicted as for TtSlyD_FL_:T1 in panel a. The peptide could be confidently modeled and, surprisingly, a *trans*-valine residue (V15_S3_) is found in place of a *cis*-proline in the center of the binding pocket (right panel). Notably, the main chain interactions of the S3 peptide with N35 and I37 are almost the same as for the S2 and S2-W23A peptides, except that the hydrogen bond with N35 is water mediated. It is furthermore noteworthy that a lysine side chain of the peptide (K16_S3_) interacts with the bound chloride ion of the FKBP domain, as is also the case for the S2 and S2-W23A peptides (K28_S2_). (PNG 1640 kb) Additional file 9: Mapping of the interaction faces between the FKBP domain and the bound peptides/FK506 for representative structures. **a** The S2-W23A peptide bound in a type VIa1 β-turn configuration to the FKBP domain of TtSlyD_FL_:S2-W23A molecule A. The peptide is shown in sticks and TtSlyD_FL_ in semi-transparent surface representation. Residues forming van der Waal interactions, hydrogen bonds, or salt bridges between TtSlyD_FL_ and the peptide are colored as in Fig. 5. All non-interacting residues are dark gray in both protein and peptide. Selected residues in the peptide are labeled. **b** The S2-W23A peptide bound in a type VIb-like β-turn configuration to TtSlyD_FL_:S2-W23A molecule D. **c** TtSlyD_FL_:FK506 molecule B. Here the whole FK506 molecule is colored pink. **d** Interaction lists for the structures shown in panels a–c. Hydrogen bonds, salt bridges, and van der Waal interactions were annotated as in Additional file 6, and the color scheme is the same as in panels a–c. Asterisks designate β-strand-type hydrogen bonds between K28_S2_ and N35/I37, or the equivalent interactions between FK506 and N35/I37. The dagger symbols designate residues that are poorly defined in the electron density map (real-space correlation coefficient < 0.7). Interactions with the chloride ion and symmetry-related mates are omitted. Note that the peptides bound to the FKBP domain also interact with the inter-domain loops and to some extent the IF domain, while peptides bound to the IF domain interact almost exclusively with that domain (Additional file 6). The table seems to imply that P29_S2_ interacts differently with the hydrophobic pocket in the type VIa1 and VIb-like binding modes. However, this is partially because several of the nearby potentially interacting residues balance around the van der Waal cut-off value. Indeed, the positions of P29_S2_ in these two binding modes overlap rather strongly (Fig. 6a, b). (PNG 2669 kb)

## Abbreviations

DMSO: Dimethyl sulfoxide
FKBP: FK506-binding protein
HEPES: 4-(2-hydroxyethyl)-1-piperazineethanesulfonic acid
IF: Insert-in-flap
IPTG: Isopropyl-β-D-thiogalactopyranoside
ITC: Isothermal titration calorimetry
NMR: Nuclear magnetic resonance
PEG: Polyethylene glycol
PPIase: Peptidyl-prolyl isomerase
SAD: Single-wavelength anomalous diffraction
suc-ALPF-pNA: succinyl-Ala-Leu-Pro-Phe-4-nitroanilide
TLS: Translation libration screw
TtSlyD: SlyD from *Thermus thermophilus*
TtSlyD_FL_: full-length TtSlyD
TtSlyD_ΔIF_: TtSlyD with the IF domain deleted (and replaced by the flap loop from human FKBP12)
TROSY-HSQC: ^1^H–^13^C transverse relaxation optimized spectroscopy hetero single quantum coherence
