# Supplementary figures and images for: Molecular insights into substrate recognition and catalytic mechanism of the chaperone and FKBP peptidyl-prolyl isomerase SlyD
Source: BMC Biol. 2016 Sep 23;14:82. doi: 10.1186/s12915-016-0300-3 (PMC5034536; doi:10.1186/s12915-016-0300-3)

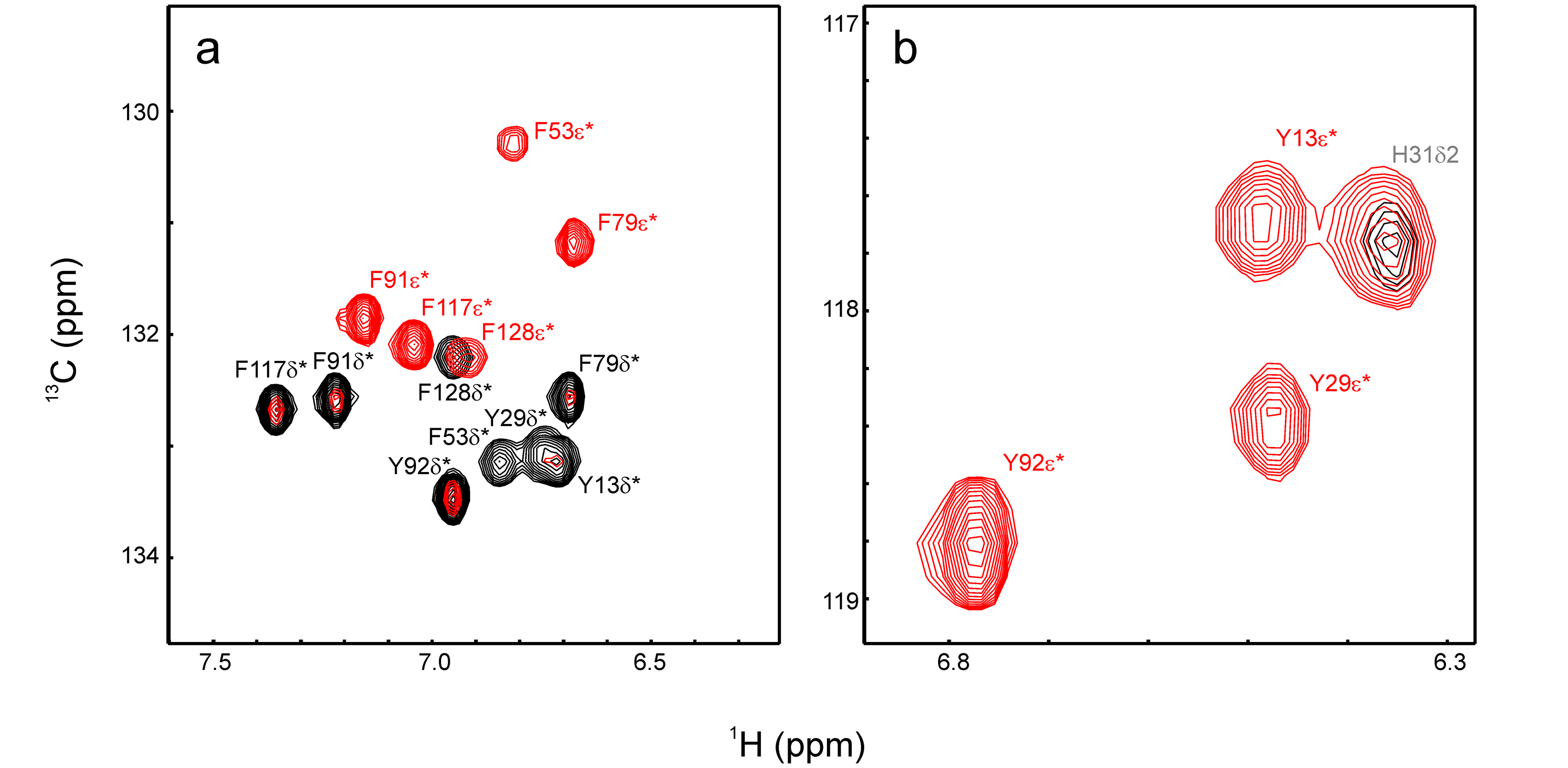

Supplement: Additional file 1: — 1H–13C transverse relaxation optimized spectroscopy hetero single quantum coherence (TROSY-HSQC) spectra of 1 mM apo TtSlyD at 25 °C. a The Fδ,ε/Yδ region. b The Yε region. δ positions are 13C-labeled by 1-13C1-glucose and shown in black. ε are 13C-labeled by 2-13C1-glucose and shown in red. All expected resonances have been identified and assigned, with the exception of Y63, which presumably is line-broadened beyond detection due to conformational exchange. (PNG 363 kb) [file 12915_2016_300_MOESM1_ESM.png]

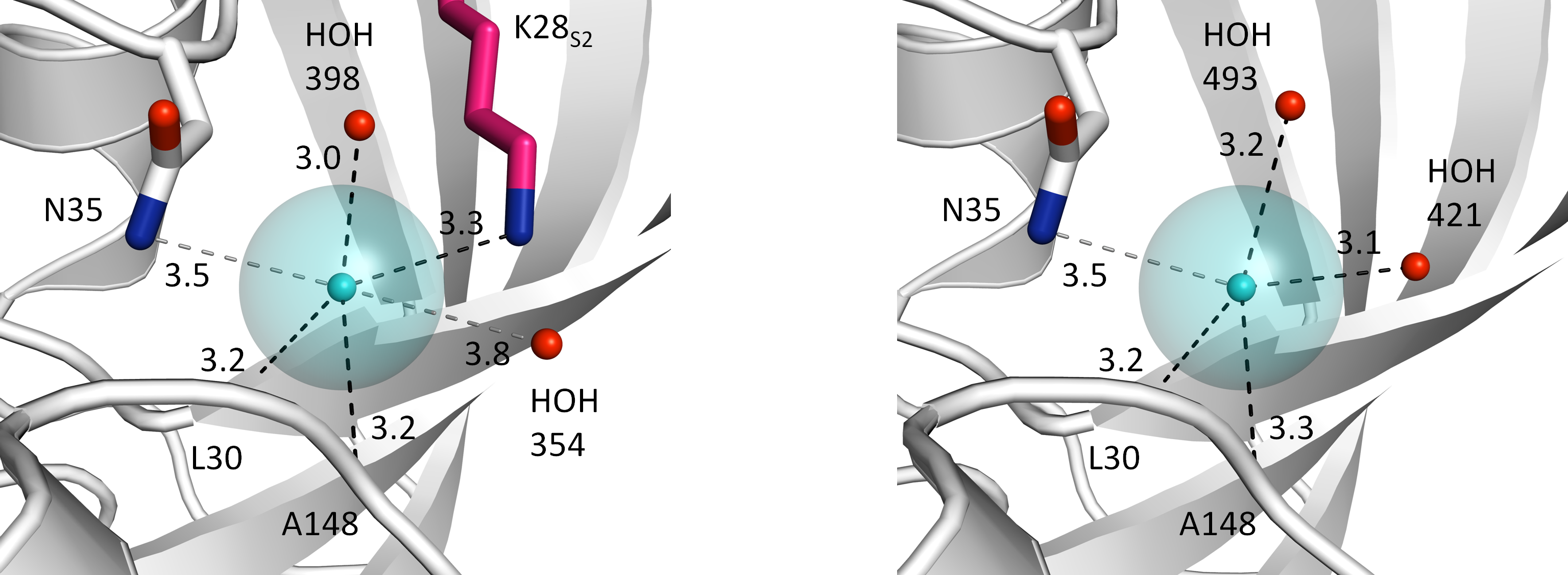

Supplement: Additional file 2: — Chloride binding site. Two examples are shown: TtSlyDFL:S2-W23A chain A (left) and TtSlyDFL:FK506 chain B (right). TtSlyD is white, the peptide is pink, chloride is turquoise, and water molecules are red. The transparent spheres indicate the van der Waal radii of the chloride ions as given by PyMol. Distances up to 3.4 Å (first coordination sphere of chloride [70]) are indicated with black dashes and longer distances are gray. The annotation as chloride was based on the binding mode and the electron density maps (including the anomalous difference Fourier map where weak density for the ion could be recognized). The backbone nitrogen atoms of L30 and A148 interact with the ion. The distance here is ~3.2 Å for all structures, which is the most commonly found coordination distance for chloride [70]. The side chain of N35 also contacts the ion, though the distances here are longer and more variable. When an S2, S2-W23A, or S3 peptide is bound, the Nζ atom of K28S2/K16S3 is within 3.1–3.6 Å of the ion (left panel). The number of ligands is typically three to five, which is also the most common range for chloride in general [70]. Part of the coordination sphere is made up of water molecules that vary somewhat in exact position. Note that the chloride ion is present under both low and moderately high salt concentrations (Table 5), indicating that it is unlikely to be a crystallization artifact. (PNG 975 kb) [file 12915_2016_300_MOESM2_ESM.png]

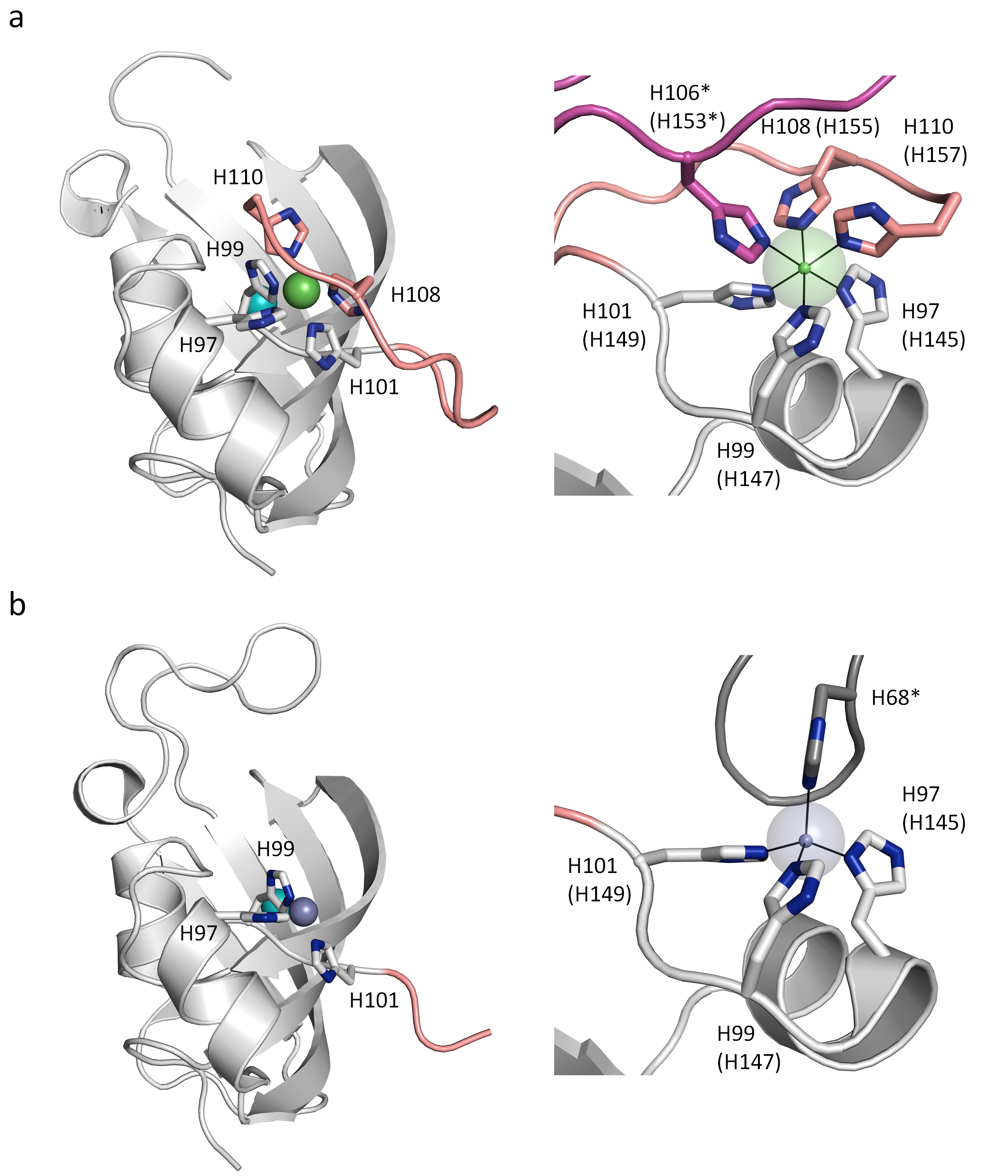

Supplement: Additional file 3: — Metal coordination. Binding of a nickel ion in TtSlyDΔIF:S2-W23A a and a zinc ion in TtSlyDΔIF:FK506 chain A b. Native protein residues are white and the His-tag is pale pink, except for symmetry-related mates where the protein is dark gray and the His-tag is purple. Chloride is turquoise, the putative nickel ion is green, and the zinc ion is slate. Two views are shown: an overview in the same orientation as in Fig. 2 (left panels) and a zoomed view, which is reoriented to better show the coordination geometry (right panels). In the latter view, coordination bonds are indicated with solid black lines and residues are labeled (the corresponding residues of the TtSlyDFL construct are indicated in parentheses). Asterisks denote residues in symmetry-related mates. The putative nickel ion is octahedrally coordinated by six histidine residues: three native residues, two from the His-tag, and one from the His-tag of a symmetry-related mate. The same coordination pattern is seen in TtSlyDΔIF:S3 and in a previously determined structure of TtSlyDFL [PDB: 3CGM] [17]. The zinc ion is tetrahedrallly coordinated by the same three native histidines residues that are also used for coordinating the putative nickel ion, as well as a histidine from a symmetry-related mate. A similar pattern is seen in another of the previously determined TtSlyDFL structures [PDB: 3LUO] [17], though here the symmetry-related histidine residue is replaced by a water molecule, which is bound at a somewhat different angle. (PNG 2098 kb) [file 12915_2016_300_MOESM3_ESM.png]

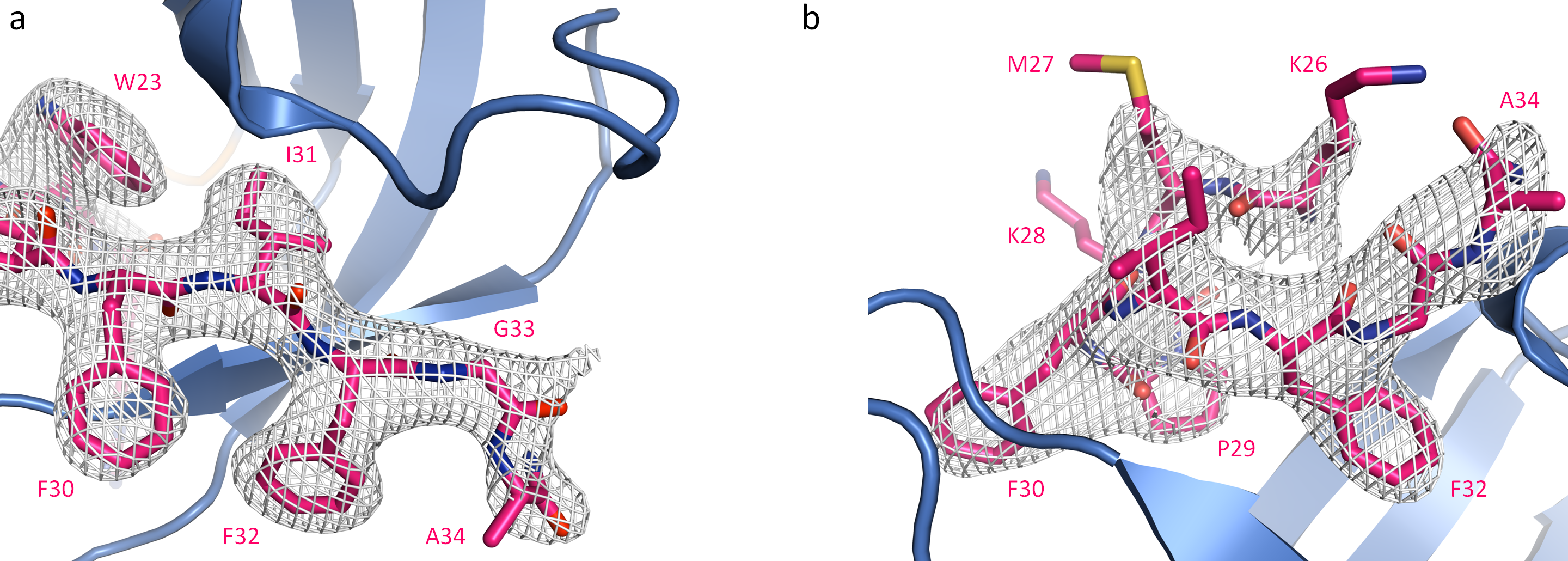

Supplement: Additional file 4: — Electron density maps for the S2 peptide bound to the IF domain. a Electron density map for the S2 peptide bound to the IF domain of molecule A of the 2.9-Å TtSlyDFL:S2 structure. The IF domain is blue, the S2 peptide is pink, and the 2Fo-Fc electron density map for the peptide is gray (contoured at 1 σ). In spite of the modest resolution, the peptide is well defined in the electron density map, and could therefore be confidently modeled. b The S2 peptide bound to molecule B in the TtSlyDFL:S2 structure. This peptide is not very well defined in the electron density map, and could therefore not be modeled with high confidence. However, it is nonetheless clear that it is bound in a very different manner than the peptide bound to molecule A (see also Fig. 4b, c). (PNG 2247 kb) [file 12915_2016_300_MOESM4_ESM.png]

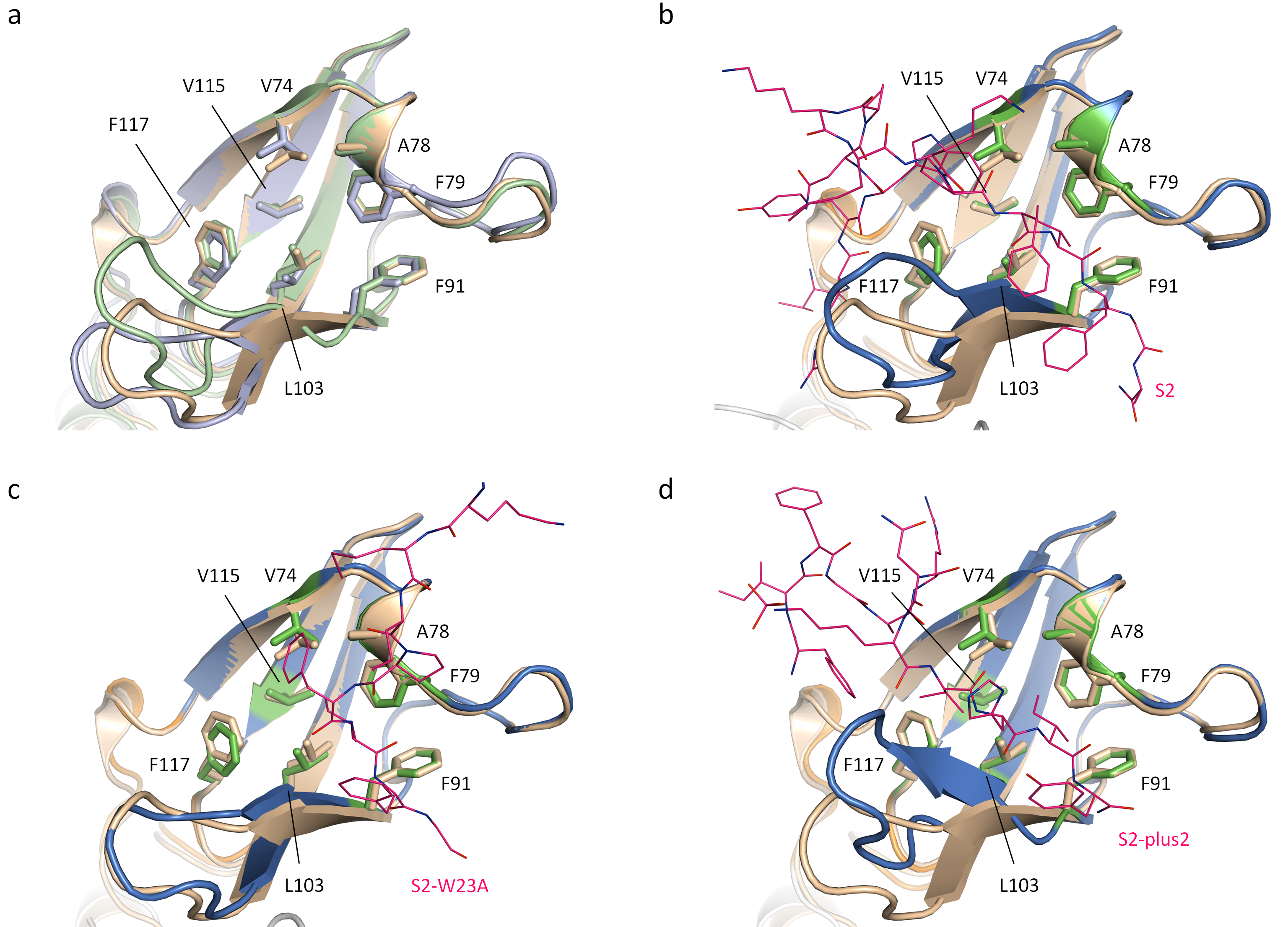

Supplement: Additional file 5: — Structural changes in the IF domain upon substrate binding. a Overlay of the IF domain of the three TtSlyDFL molecules of the TtSlyDFL:FK506 structure, which is the only structure we obtained where no substrate was bound to the IF domain. Molecule A is pale green, molecule B is wheat, and molecule C is pale slate. The side chains of residues lining the hydrophobic groove are shown in sticks. Note that there is very little variation in the positions and conformations of these residues, except that some variability can be observed for V74, L103, and F117. b–d Overlay of the substrate-free IF domain of TtSlyDFL:FK506 molecule B with three substrate-bound structures: TtSlyDFL:S2 molecule A (panel B), TtSlyDFL:S2-W23A molecule C (panel C), and S2-plus2 (panel D). TtSlyDFL:FK506 molecule B is colored wheat, as in panel C, and the substrate-bound structures are colored as in Fig. 4. Binding does not notably affect the hydrophobic binding groove. Thus, only V74, L103, and F117 display clear variability between the substrate-bound structures and the TtSlyDFL:FK506 molecule B apo structure, and not more pronouncedly so than between the three individual TtSlyDFL molecules in the TtSlyDFL:FK506 apo structure (panel A). (PNG 2763 kb) [file 12915_2016_300_MOESM5_ESM.png]

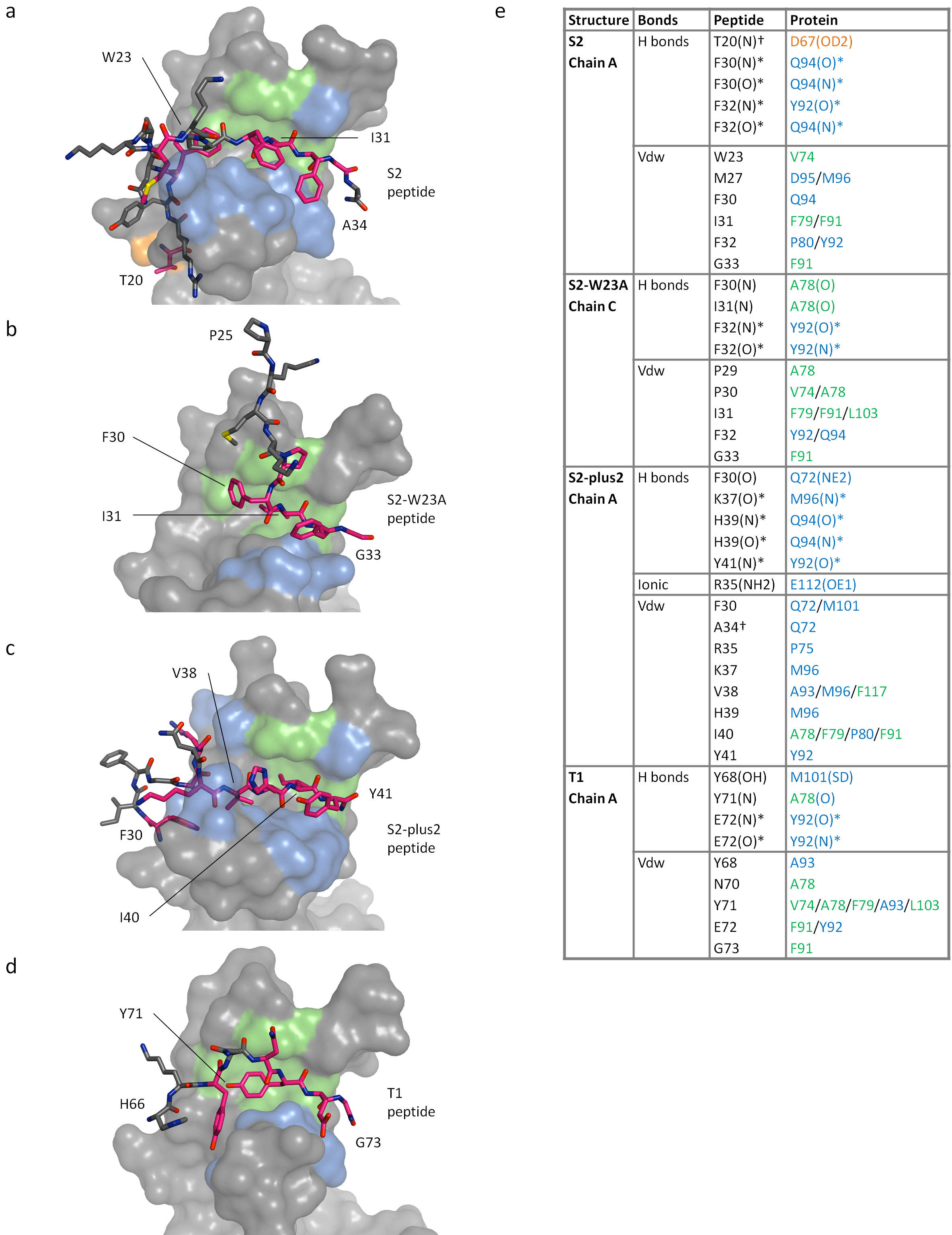

Supplement: Additional file 6: — Mapping of the interaction faces between the IF domain and the bound peptides for representative structures. a The S2 peptide bound to the IF domain of molecule A in the TtSlyDFL:S2 structure. The peptide is shown in sticks and TtSlyDFL in semi-transparent surface representation. Residues forming van der Waal interactions, hydrogen bonds, or salt bridges between TtSlyD and the peptide are colored as in Fig. 4, while all non-interacting residues are dark gray in both protein and peptide. Selected residues in the peptide are labeled. b The S2-W23A peptide bound to the IF domain of molecule C in the TtSlyDFL:S2-W23A structure. c The S2-plus2 peptide bound to the IF domain in the TtSlyDFL:S2-plus2 structure. d The T1 peptide bound to the IF domain in the TtSlyDFL:T1 structure. e Interaction lists for the structures shown in panels a–d. Hydrogen bonds (≤3.5 Å) and salt bridges (≤4.0 Å) detected using the PISA program are listed along with van der Waal interactions between carbon and carbon/sulfur atoms (≤4.0 Å). The specific atoms mediating the interactions between residues are specified for the hydrogen bonds and salt bridges. Interacting residues are colored as in panel a–d. Asterisks designate β-strand hydrogen bonds augmenting the β8–β9 hairpin, and dagger symbols designate residues that are poorly defined in the electron density map (real-space correlation coefficient < 0.7). Interactions with symmetry-related mates are omitted. There are few hydrogen bonds and salt bridges apart from the β-strand interactions, while there are many peripheral van der Waal interactions that do not involve the hydrophobic groove, most of which involve the β8–β9 hairpin instead. (PNG 1947 kb) [file 12915_2016_300_MOESM6_ESM.png]

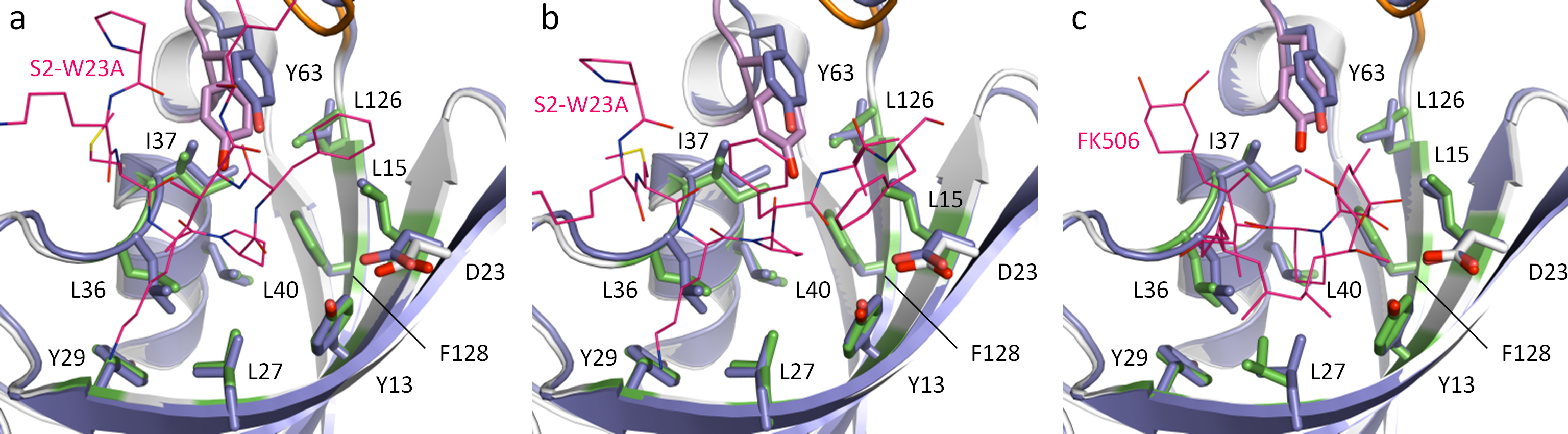

Supplement: Additional file 7: — Structural changes in the FKBP domain upon substrate binding. a Superimposition of molecule A from the TtSlyDFL:S2-W23A structure, which represents the type VIa1 binding mode, on the FKBP domain of TtSlyDFL:S2-plus2, which represents the substrate-free form. The substrate-bound structure is colored as in Fig. 5 and the apo structure is colored slate. b Superimposition of molecule D from the TtSlyDFL:S2-W23A structure, which represents the type VIb-like binding mode, on the apo form (same color scheme as in panel a). c Superimposition of molecule B from the TtSlyDFL:FK506 structure, which represents the FK506 binding mode on the apo form (same color scheme as in panel a). Overall, there is substantial variation in the positions of Y63, and a more modest variation in the position of the loop encompassing L36 and I37 between the substrate-bound forms and the apo form. There is no overall positional shift in any of the other residues in the hydrophobic binding pocket, though some variability in the side chain configurations of D23, L27, and L126 can be detected, which may or may not be linked to substrate binding. (PNG 1394 kb) [file 12915_2016_300_MOESM7_ESM.png]

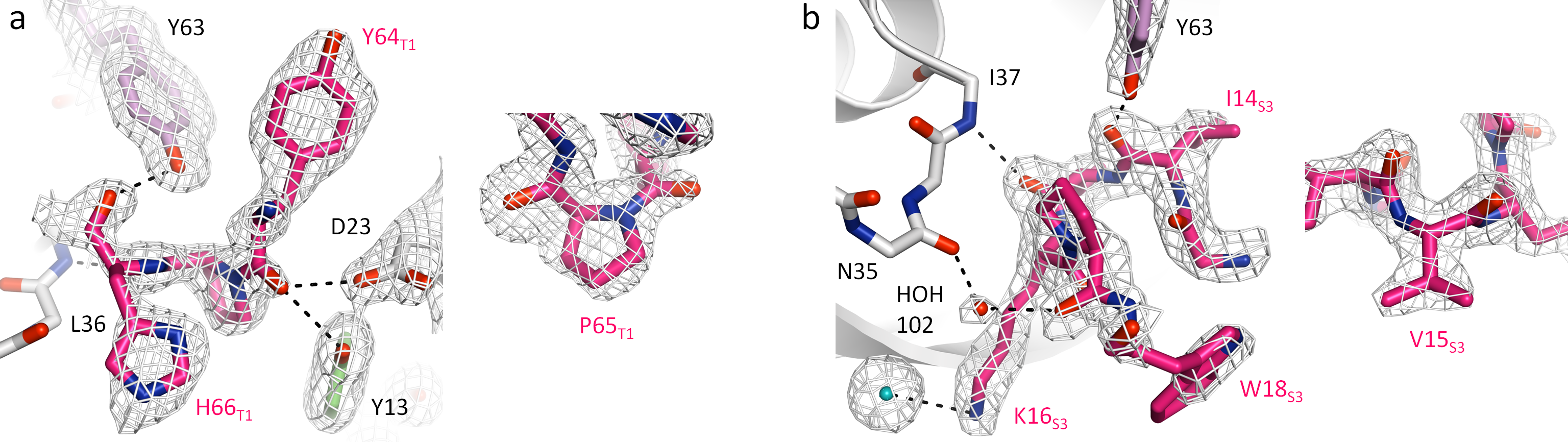

Supplement: Additional file 8: — Non-canonical binding to the FKBP domain. a Binding of the T1 peptide to the FKBP domain in the 1.6-Å structure of TtSlyDFL:T1. The model is colored as in Fig. 5, and the 1 σ 2Fo-Fc electron density map is shown in gray around the peptide and key binding site residues. Two views are shown – an overview displaying putative hydrogen bonds (black dashes) on the left and a focused view of P65 of the peptide (P65T1) on the right. Although the electron density map is generally of high quality for this structure (as exemplified by Y63 in the left panel), it is quite poor for the peptide bound to the FKBP domain, suggesting that the occupancy is low and/or the flexibility is high. This agrees well with the binding studies, which showed that the T1 peptide binds with low affinity. Nonetheless, P65T1 could be placed fairly confidently in the center of the binding site where it is bound in the cis form. Notably, it is bound in the opposite direction as compared to the S2 and S2-W23A peptides. b Binding of the S3 peptide to the FKBP domain in the 2.0-Å structure of TtSlyDΔIF:S3. Depicted as for TtSlyDFL:T1 in panel a. The peptide could be confidently modeled and, surprisingly, a trans-valine residue (V15S3) is found in place of a cis-proline in the center of the binding pocket (right panel). Notably, the main chain interactions of the S3 peptide with N35 and I37 are almost the same as for the S2 and S2-W23A peptides, except that the hydrogen bond with N35 is water mediated. It is furthermore noteworthy that a lysine side chain of the peptide (K16S3) interacts with the bound chloride ion of the FKBP domain, as is also the case for the S2 and S2-W23A peptides (K28S2). (PNG 1640 kb) [file 12915_2016_300_MOESM8_ESM.png]

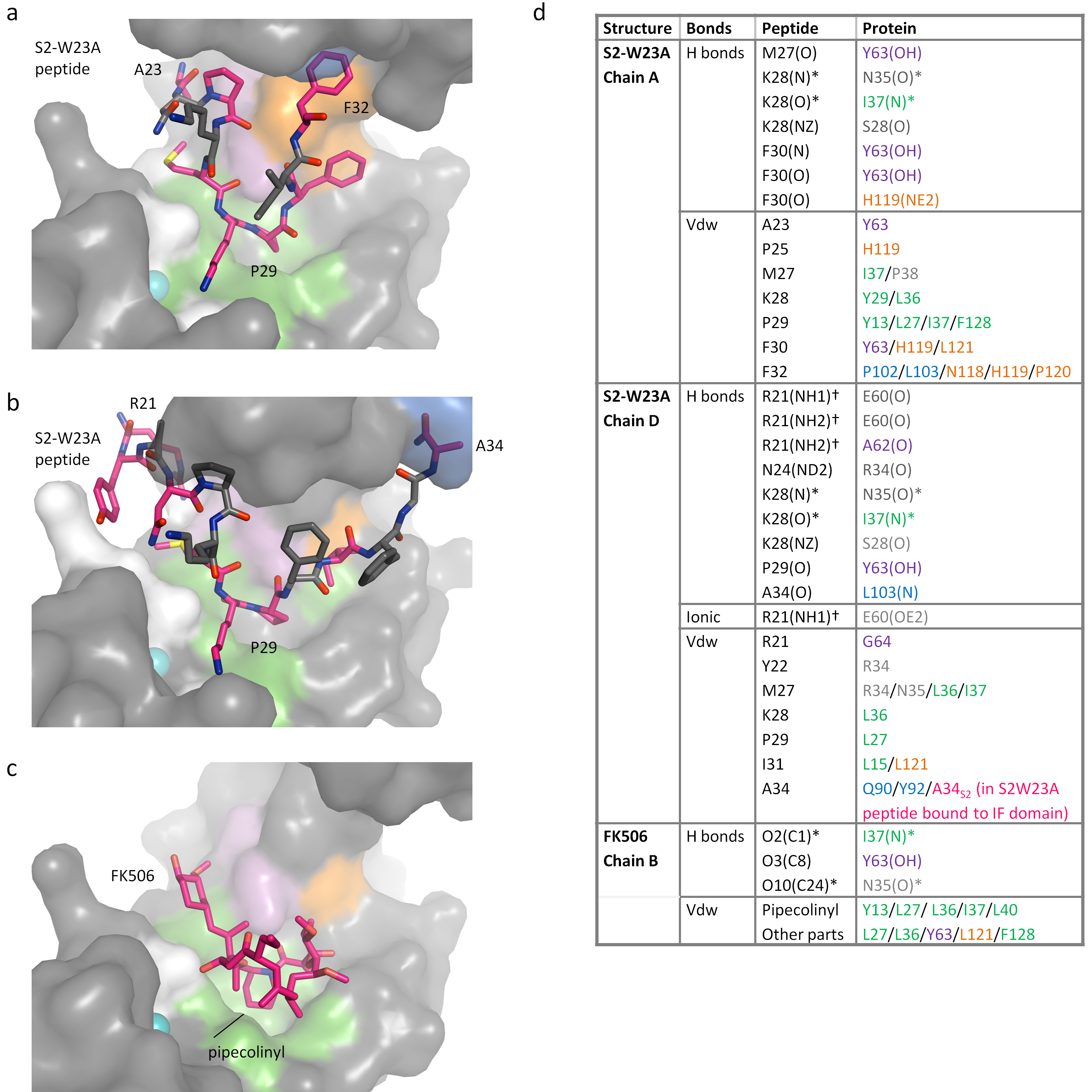

Supplement: Additional file 9: — Mapping of the interaction faces between the FKBP domain and the bound peptides/FK506 for representative structures. a The S2-W23A peptide bound in a type VIa1 β-turn configuration to the FKBP domain of TtSlyDFL:S2-W23A molecule A. The peptide is shown in sticks and TtSlyDFL in semi-transparent surface representation. Residues forming van der Waal interactions, hydrogen bonds, or salt bridges between TtSlyDFL and the peptide are colored as in Fig. 5. All non-interacting residues are dark gray in both protein and peptide. Selected residues in the peptide are labeled. b The S2-W23A peptide bound in a type VIb-like β-turn configuration to TtSlyDFL:S2-W23A molecule D. c TtSlyDFL:FK506 molecule B. Here the whole FK506 molecule is colored pink. d Interaction lists for the structures shown in panels a–c. Hydrogen bonds, salt bridges, and van der Waal interactions were annotated as in Additional file 6, and the color scheme is the same as in panels a–c. Asterisks designate β-strand-type hydrogen bonds between K28S2 and N35/I37, or the equivalent interactions between FK506 and N35/I37. The dagger symbols designate residues that are poorly defined in the electron density map (real-space correlation coefficient < 0.7). Interactions with the chloride ion and symmetry-related mates are omitted. Note that the peptides bound to the FKBP domain also interact with the inter-domain loops and to some extent the IF domain, while peptides bound to the IF domain interact almost exclusively with that domain (Additional file 6). The table seems to imply that P29S2 interacts differently with the hydrophobic pocket in the type VIa1 and VIb-like binding modes. However, this is partially because several of the nearby potentially interacting residues balance around the van der Waal cut-off value. Indeed, the positions of P29S2 in these two binding modes overlap rather strongly (Fig. 6a, b). (PNG 2669 kb) [file 12915_2016_300_MOESM9_ESM.png]
